# Supplementary material for: Density physics-informed neural networks reveal sources of cell heterogeneity in signal transduction
Source: Patterns (N Y). 2023 Dec 26;5(2):100899. doi: 10.1016/j.patter.2023.100899 (PMC10873160; doi:10.1016/j.patter.2023.100899)
Supplement: Document S1. Notes S1–S7, Figures S1–S6, and Tables S1 and S2 [file mmc1.pdf]

Patterns, Volume *5*

Supplemental information

Density physics-informed neural networks reveal sources of cell heterogeneity in signal transduction

Hyeontae Jo, Hyukpyo Hong, Hyung Ju Hwang, Won Chang, and Jae Kyoung Kim

SUPPLEMENTAL INFORMATION

Supplemental Notes

Note S1. Differential equation describing the mean of a stochastic delayed birth-death process

A stochastic delayed birth-death process can be used to describe a cell signaling pathway that consists of signal activation, transduction, and final response (Figure 1A). Specifically, signal activation is represented by a birth reaction with a rate of λ_b, the activated signal undergoes a delay with density $g(t)$, and the decay of the final response molecules is represented by a death reaction with a rate of λ_d. According to the Transient Little's law,[1] the mean time trace of the stochastic process $y(t)$, is given by

$$y(t) = \int_0^t \lambda_b G(t-\tau)\exp(-\lambda_d \tau)\, d\tau = \lambda_b (G * h)(t),$$

where $G(t) = \int_0^t g(s)ds$ and $h(t) = \exp(-\lambda_d t)$ for $t \geq 0$ and $h(t) = 0$ for $t < 0$, which represents a survival probability of the response molecules after time t (see Kim et al.[2] for details). To derive the differential equation for $y(t)$, we took a derivative and got the following:

$$y'(t) = \lambda_b (G * h')(t) = \lambda_b (G * [\delta_0(\tau) + (-\lambda_d) h])(t) = \lambda_b G(t) - \lambda_d \lambda_b (G * h)(t)$$

where $\delta_0(\tau)$ is the Dirac delta measure at time 0. Finally, we obtained the differential equation that the mean time trace $y(t)$ satisfies as follows:

$$y'(t) = \lambda_b \int_0^t g(s)ds - \lambda_d y(t).$$

This differential equation is used to form a physics loss for our inference model (Figure 2B).

Note S2. Minimizing the data and physics losses ensures the convergence of an approximated transduction-time distribution, $\tilde{g}(t)$, to the true transduction-time distribution, $g(t)$.

We use $\tilde{y}(t) = \sum_{j=1}^{M} \omega_j' \int_0^t K(u; c_j, s_j)du$ instead of the exact solution of Equation 1, $\tilde{y}_{ex}(t) = \lambda_b \sum_{j=1}^{M} \omega_j e^{-\lambda_d t} * \int_0^t K(u; c_j, s_j)du$ for computational efficiency (Figure 2). Here, we justify this by showing that even when $\tilde{y}(t)$ is used, $\tilde{g}$ converges to g when $\tilde{y}(t)$ minimizes both data and physics losses simultaneously. To do this, we prove the following two steps.

Step 1: $\tilde{y}_{ex}(t)$ converges to y when $\tilde{y}(t)$ minimizes both data and physics losses.
Step 2: $\tilde{g}$ converges to g when $\tilde{y}_{ex}$ converges to y.

Proof of Step 1: We indirectly quantify the difference between $\tilde{y}_{ex}$ and $\tilde{y}$ using the physics loss. First, let $p(t)$ be the function given by substituting $\tilde{y}$ into Equation 1.

$$p(t) \coloneqq \frac{d\tilde{y}}{dt} + \lambda_d \tilde{y} - \lambda_b \int_0^t \tilde{g}(u)du.$$

Since $\tilde{y}_{ex}$ exactly satisfies the equation $\frac{d\tilde{y}_{ex}}{dt} + \lambda_d \tilde{y}_{ex} = \lambda_b \int_0^t \tilde{g}(u)du$, we can replace $\int_0^t \tilde{g}(u)du$ with $\frac{d\tilde{y}_{ex}}{dt} + \lambda_d \tilde{y}_{ex}$ as follows:

$$p(t) = \frac{d}{dt}(\tilde{y} - \tilde{y}_{ex}) + \lambda_d(\tilde{y} - \tilde{y}_{ex}).$$

By solving the differential equation with respect to $\tilde{y} - \tilde{y}_{ex}$, we obtain the following formula:

$$\tilde{y} - \tilde{y}_{ex} = e^{-\lambda_d t} * p(t).$$

By taking $L^1([0,T])$-norm on both sides and using the Young's convolution inequality, we obtain

$$\|\tilde{y} - \tilde{y}_{ex}\|_{L^1([0,T])} \leq \left\|e^{-\lambda_d t}\right\|_{L^1([0,T])} \|p(t)\|_{L^1([0,T])}.$$

Thus, $\tilde{y}$ converges to $\tilde{y}_{ex}$ by minimizing the physic loss, $\|p(t)\|_{L^1([0,T])}$. Finally, using the triangle inequality,

$$\|\tilde{y}_{ex} - y\|_{L^1([0,T])} \leq \|\tilde{y}_{ex} - \tilde{y}\|_{L^1([0,T])} + \|\tilde{y} - y\|_{L^1([0,T])}$$

$$\leq \left\| e^{-\lambda_d t} \right\|_{L^1([0,T])} \text{physics loss} + \text{data loss},$$

we can conclude that $\tilde{y}$ can closely approximate $\tilde{y}_{ex}$ by simultaneously minimizing both data and physics losses. We also showed this using numerical optimization (Figure S1).

Proof of Step2: y and $\tilde{y}_{ex}$ satisfy Equation 1 corresponding to g and $\tilde{g}$, respectively. That is,

$$\frac{d\tilde{y}_{ex}}{dt} + \lambda_d \tilde{y}_{ex} = \lambda_b \int_0^t \tilde{g}(u)du,$$

$$\frac{dy}{dt} + \lambda_d y = \lambda_b \int_0^t g(u)du.$$

By substituting both sides and taking $L^1([0,T])$-norm, we can derive the following inequality:

$$\lambda_b \left\| \int_0^t \left(\tilde{g}(u) - g(u)\right)du \right\|_{L^1([0,T])} = \|(\tilde{y}_{ex} - y)' + \lambda_d(\tilde{y}_{ex} - y)\|_{L^1([0,T])}$$

$$\leq \|(\tilde{y}_{ex} - y)'\|_{L^1([0,T])} + \lambda_d \|\tilde{y}_{ex} - y\|_{L^1([0,T])}.$$

We then aim to show $\|(\tilde{y}_{ex} - y)'\|_{L^1([0,T])}$ is close to 0 when $\|\tilde{y}_{ex} - y\|_{L^1([0,T])}$ is close to 0. Let $\tilde{y}_{ex,n}$ and $\tilde{g}_n$ denote the trained functions at n-th iteration when we minimize $\|\tilde{y}_{ex} - y\|_{L^1([0,T])}$, i.e., $\tilde{g}_n = \sum_{j=1}^{M} \omega_{j,n} K\left(t; c_{j,n}, s_{j,n}\right)$, for $n = 1,2, \ldots$. It is clear that $\tilde{y}_{ex,n}$ is bounded by the steady state solution $\frac{\lambda_b}{\lambda_d}$, i.e., $\tilde{y}_{ex,n}(t) \leq \frac{\lambda_b}{\lambda_d}$, for any $\tilde{g}_n$ and time $t \in [0,T]$. Then, we obtain the following upper bound for $\tilde{y}'_{ex,n}(t)$:

$$\left|\tilde{y}'_{ex,n}(t)\right| \leq \lambda_d \left|\tilde{y}_{ex,n}(t)\right| + \lambda_b \left| \int_0^t \tilde{g}_n(u)du \right| \leq \lambda_d \frac{\lambda_b}{\lambda_d} + \lambda_b = 2\lambda_b.$$

By substituting the differential equations for $\tilde{y}_{ex,n}(t)$ and $\tilde{y}_{ex,n}(v)$, for $t, v \in [0,T]$, we can derive the following inequality:

$$\left|\tilde{y}'_{ex,n}(t) - \tilde{y}'_{ex,n}(v)\right| \leq \lambda_d \left|\tilde{y}_{ex,n}(t) - \tilde{y}_{ex,n}(v)\right| + \lambda_b \left| \int_v^t \tilde{g}_n(u)du \right|$$

$$\leq 2\lambda_b \lambda_d |t - v| + \lambda_b C(s_1, \ldots, s_M)|t - v|,$$

where the first term in the last inequality is obtained by the mean-value theorem, and the $C(s_1, \ldots, s_M)$ is the Lipschitz constant for $\int_v^t \tilde{g}_n(u)du$. Since $C(s_1, \ldots, s_M)$ is independent of (n, t, v) and s_j in $C(s_1, \ldots, s_M)$ is contained in $[s_{min}, s_{max}]$, $\left|\tilde{y}'_{ex,n}(t)\right|$ is Lipschitz continuous with a constant $C = 2\lambda_b \lambda_d + \lambda_b C(s_1, \ldots, s_M)$. That is,

$$\left|\tilde{y}'_{ex,n}(t) - \tilde{y}'_{ex,n}(v)\right| \leq C|t - v|,$$

which implies that $\left\{\tilde{y}_{ex,n}\right\}_{n=1}^{\infty}$ is equicontinuous. Since $\left\{\tilde{y}'_{ex,n}\right\}_{n=1}^{\infty}$ is uniformly bounded and equicontinuous, we can extract a uniformly convergent subsequence by the Arzela-Ascoli theorem, and its limit converges to y'. In summary, if $\tilde{y}_{ex}$ converges to y during the training step, $\int_0^t \tilde{g}(u)du$ converges to $\int_0^t g(u)du$. Consequently, $\tilde{g}(t)$ converges to $g(t)$ by differentiating Equation 1 and by repeating same procedure.

Note S3. $\|s(\boldsymbol{\theta}_{\text{enc}}, \boldsymbol{\theta}_{\text{dec}})\|$ in the regularization loss ensures the smoothness of an estimated transduction-time distribution $\tilde{g}(t)$.

In the regularization loss $\mathcal{L}_r(\theta) = \|s(\theta_{\text{enc}}, \theta_{\text{dec}})\| + D_{\text{KL}}\left(q_{\theta_{\text{enc}}}(\boldsymbol{z}|\boldsymbol{y})||N(\boldsymbol{0}, \boldsymbol{I})\right)$ (Figure 2B), the first term is given by

$$\|s(\theta_{\text{enc}}, \theta_{\text{dec}})\| = \frac{1}{Nd} \sum_{i=1}^{N} \sum_{j=1}^{d} \left| s_i(t_j) - \left(\beta_i(t_j) s_{\min} + \left(1 - \beta_i(t_j)\right) s_{\max}\right) \right|,$$

where $\beta_i(t_j) = \frac{\frac{d\langle y_i \rangle_+}{dt}(t_j) - \min \frac{d\langle y_i \rangle_+}{dt}(t)}{\max \frac{d\langle y_i \rangle_+}{dt}(t) - \min \frac{d\langle y_i \rangle_+}{dt}(t)}$ and $s_{\min}$ and $s_{\max}$ are the lower and upper bounds of the scale parameters, respectively. This term ensures the smoothness of an estimated transduction-time distribution $\tilde{g}(t)$ by preventing too small or large values of the scale parameters. Specifically, when

a time trace $\boldsymbol{y}_i$ dramatically changes, i.e., $\frac{d\langle \boldsymbol{y}_i\rangle_+}{dt}(t_j) \approx \max \frac{d\langle \boldsymbol{y}_i\rangle_+}{dt}(t)$, we get $\beta_i(t_j) \approx 1$ and thus $s_i(t_j) \approx s_{\min}$. That is, when there is a dramatic change in $\boldsymbol{y}_i$, the scale parameters are set to be small and thus the width of the kernel at t_j becomes narrow. On the other hand, when the time trace $\boldsymbol{y}_i$ is nearly flat, i.e., $\frac{d\langle \boldsymbol{y}_i\rangle_+}{dt}(t_j) \approx \min \frac{d\langle \boldsymbol{y}_i\rangle_+}{dt}(t)$, we get $\beta_i(t_j) \approx 0$, and hence $s_i(t_j)$ is penalized if it is far from $s_{\max}$. That is, when there is no dramatic change in $\boldsymbol{y}_i$, we encourage the scale parameters to be large so that the kernel with a large width is used. In this way, we can avoid the use of sharp kernels, which prevents redundant fluctuation in the $\tilde{g}(t)$. That is, with the regularization term $\|s(\theta_{\text{enc}}, \theta_{\text{dec}})\|$, we can get smooth $\tilde{g}(t)$.

Note S4. Stopping criterion for training Density-PINN.

We trained the model using the Adam optimizer[3] with learning rate = 10^{-4}. In order to prevent overfitting issues, we applied an early stopping criterion[4]. During the training process, we generated 1000 samples of jth weight $\omega(c_j)$ from $p_{\theta_{\text{dec}}}\left(\boldsymbol{\omega} | q_{\theta_{\text{enc}}}(\boldsymbol{z}|\bar{\boldsymbol{y}})\right)$ at l^{th} epoch where $\bar{\boldsymbol{y}}$ is the average of time traces. We measured the average value for $\omega(c_j)$ at the lth epoch, $\bar{\omega}(c_j)[l]$. Subsequently, we computed the coefficient of variation for $\bar{\omega}(c_j)[l]$ among the last h epochs, i.e., from $(n-h+1)^{th}$ to n^{th} epoch. That is,

$$cv\left(\bar{\omega}(c_j)\right) \coloneqq cv\left(\bar{\omega}(c_j)\right)[n-h+1:n] = \frac{std\left(\bar{\omega}(c_j)\right)[n-h+1:n]}{mean\left(\bar{\omega}(c_j)\right)[n-h+1:n] + \varepsilon}.$$

Among $cv(\bar{\omega}(c_1)), \dots, cv(\bar{\omega}(c_M))$, if the number of $cv\left(\bar{\omega}(c_j)\right)$'s less than the threshold=0.01 reaches 99%, we stopped training the model. Note that h was set to be 30, and $\varepsilon = 10^{-5}$ was added in the denominator since some weights can be close to zero during training.

Note S5. Stochastic simulation

We used a delayed stochastic simulation algorithm[5] to generate the simulation data used to validate our method (Figure 3). Specifically, we performed simulation for the birth-death process with delays on the birth reaction completion[6]. The birth rate $\lambda_b = 200$, and the death rate $\lambda_d = 1$. For unimodal, weakly bimodal, strongly bimodal distributions, the PDF of the delay distributions are given by $f_\Gamma(t; 3{,}0.6)$, $0.7 f_\Gamma(t; 4.3{,}0.4) + 0.3 f_\Gamma(t - 1.6; 15{,}0.15)$, $0.6 f_\Gamma(t; 4.3{,}0.4) + 0.4 f_\Gamma(t - 1.6; 15{,}0.15)$ where $f_\Gamma(t; a, b)$ is the PDF of Gamma distribution with the shape parameter a and the scale parameter b.

Note S6. Dip statistic for multimodality

A multimodal distribution is a statistical distribution with more than one peak in its probability density function (PDF). The PDF of a multimodal distribution has multiple points where it transitions from increasing to decreasing or vice versa. Thus, the corresponding cumulative distribution function (CDF) of a multimodal distribution has multiple inflection points, where it changes from being convex to concave or vice versa. The magnitude of the multimodality can be quantified with the maximum distance between the given multimodal CDF F and its closest unimodal CDF, known as the dip statistic[7]:

$$Dip(F) = \inf_{G \in \mathcal{A}} \sup_x |F(x) - G(x)|,$$

where $\mathcal{A}$ is the class of all unimodal distribution functions.

Note S7. Step-by-step manual for Density-PINN.

We have developed a computational package implementing Density-PINN that infers delay distributions in stochastic processes. The Python code, written in Jupyter, is available at https://Github.com/mathbiomed/Density-PINN (This link will be public upon acceptance, please see the submitted code files.)

Step 1. Upload time trace data CSV file to the 'data' folder. The first column of the CSV file should contain observation time points, and the column name should be entered in the first row. For the nth row ($n \geq 2$), fill in the time series data with the name of each column. An example input file named 'sample_data.csv' is included in the 'data' folder for testing our model in Figure 3B. Ensure that the name of each column is unique.

Step 2. Open 'Density-PINN.ipynb' using Jupyter notebook. In the second cell, specify all the hyperparameters listed in Table S2 and set the 'data_name' variable to the name of your input CSV file. After making these changes, run the code by clicking 'Restart and Run All'. The trained model will be saved in the 'model' folder with the same name as the CSV file, and the program will automatically close upon completion of training.

(optional). Customize your own governing equation or target variable by editing 'utils.py' file Line 66. This file contains the model architecture and optimization code implemented using *PyTorch*.

Step 3. Run 'Visualization.ipynb'. The below output file in 'result' folder will be automatically created.

'estimation.png' visualizes an estimated transduction-time distribution and parameters, λ_b and λ_d. It also provides a comparison between the mean of time traces and the estimated $\tilde{y}(t)$ from the trained Density-PINN.

Supplemental Figures

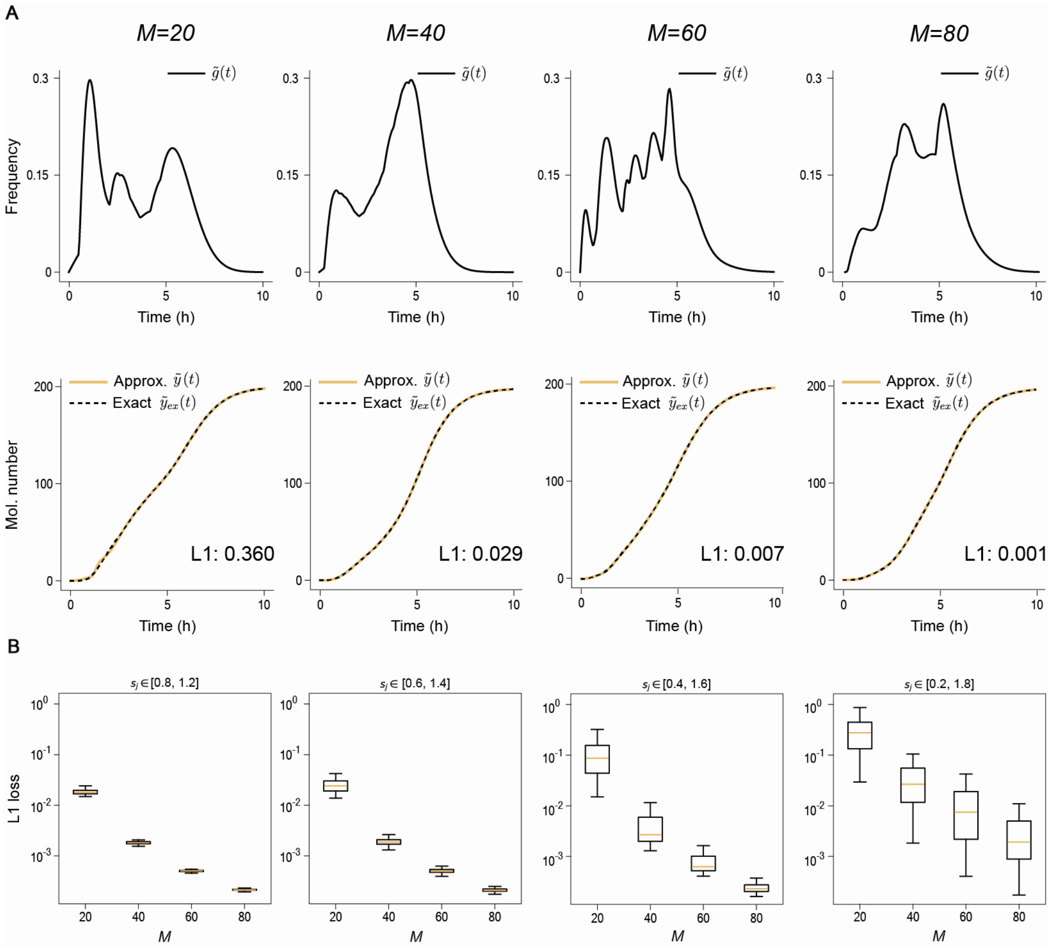

Figure S1. An approximate solution $\tilde{y}(t)$ can accurately estimate an exact solution $\tilde{y}_{ex}(t)$ of Equation 1 with $\tilde{g}(t)$. (A) We generated $\tilde{g}(t) \coloneqq \sum_{j=1}^{M} \omega_j K\left(t; c_j, s_j\right)$ using M shifted Rayleigh kernels with the shift and scale parameters c_j and s_j, respectively, and the weights ω_j. Here, $c_j = \frac{10}{2(M-1)}(j-1)$, s_j and ω_j were uniformly sampled from [0.2, 1.8] and [0,1], respectively, and ω_j were normalized so that their sum is one (top). For a given $\tilde{g}(t)$, we constructed the corresponding exact solution $\tilde{y}_{ex}(t) = \lambda_b \sum_{j=1}^{M} \omega_j e^{-\lambda_d t} * \int_0^t K\left(u; c_j, s_j\right) du$ of Equation 1 and calculated ω_j' for $\tilde{y}(t) = \sum_{j=1}^{M} \omega_j' \int_0^t K\left(u; c_j, s_j\right) du$ using the Levenberg-Marquardt method so that $\tilde{y}(t)$ approximates $\tilde{y}_{ex}(t)$ (bottom). The accuracy of $\tilde{y}(t)$ in approximating $\tilde{y}_{ex}(t)$ is quantified with the L1 loss using 100 time points evenly space in the time domain $[0, 10]$. As M increases, $\tilde{y}(t)$ more accurately approximates $\tilde{y}(t)$. (B) We repeated this process 100 times for various range of the scale parameters: $s_j \in$ [0.8, 1.2], [0.6, 1.4], [0.4, 1.6], or [0.2, 1.8]. The L1 loss between $\tilde{y}_{ex}(t)$ and $\tilde{y}(t)$ decreased as M increased in all cases.

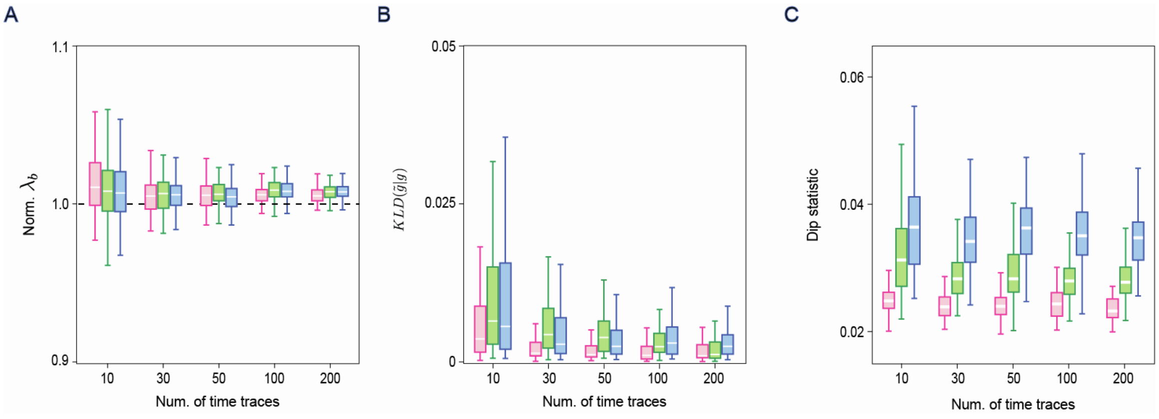

Figure S2. Density-PINN provides more accurate estimates of the activation rates and transduction-time distributions when the decay rate is fixed to its true value compared with when the decay rate is also estimated (Figure 3). (A-C) As more time traces were used for the inference, the estimation becomes more accurate: estimations of the λ_b become more accurate and precise (A) the KL-divergence between the underlying and reconstructed transduction-time distributions decreases (B), and the dip statistic, which increases as the bimodality increases, becomes more clearly distinguished among the unimodal (red), weakly bimodal (green), and strongly bimodal (blue) distributions (C).

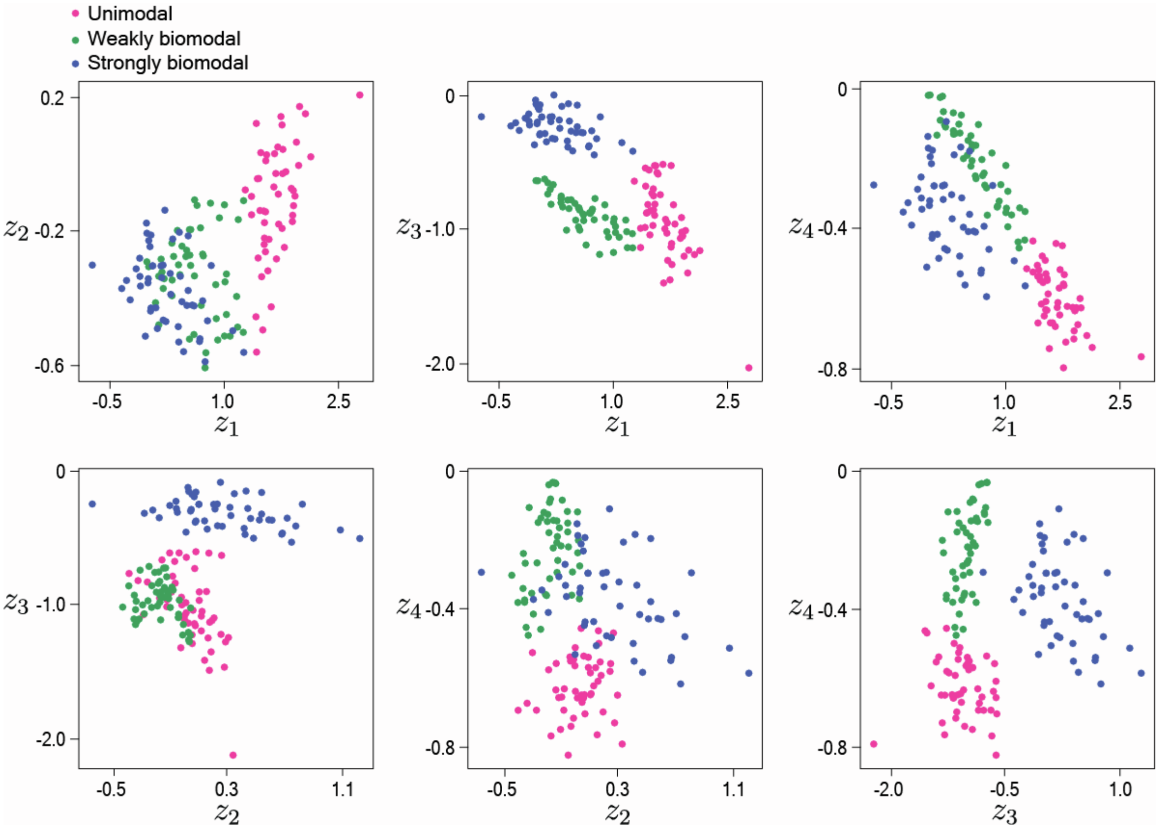

Figure S3. Visualization of latent variables corresponding to the time traces in Figure 3B. Density-PINN was trained using all time traces in Figure 3B. The resulting 4D latent variable, $\boldsymbol{z} = (z_1, z_2, z_3, z_4)$, was visualized by projecting into two dimensional spaces. The latent variables corresponding to time traces from three different types of transduction-time distributions were depicted as different colored dots: unimodal (red), weakly bimodal (green), and strongly bimodal (blue). The clear separation of the dots with different colors indicates that the Density-PINN can distinguish time traces from different transduction-time distributions.

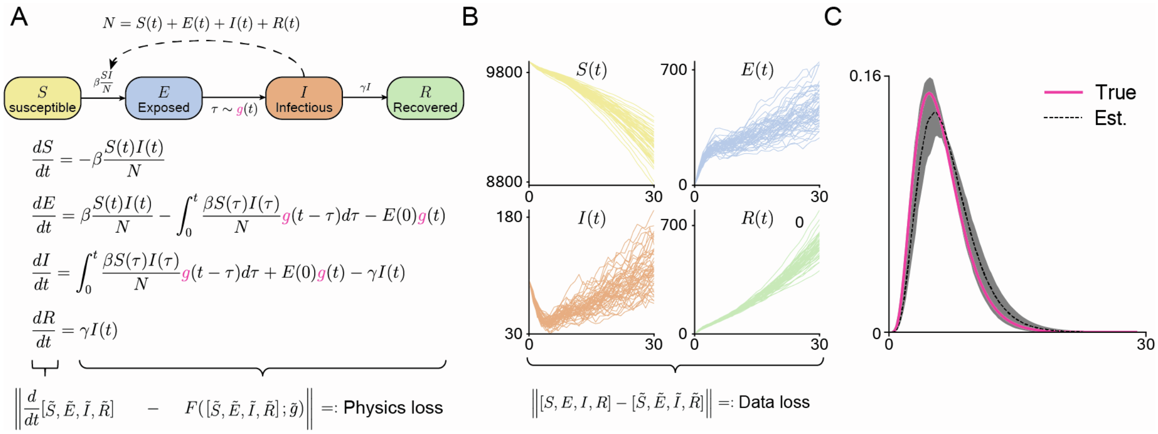

Figure S4. Density-PINN accurately estimates the latent period distribution of the SEIR Model. (A) The Susceptible-Exposed-Infectious-Recovered (SEIR) compartmental model with the latent period distribution $g(t)$ (i.e., the sojourn time in the compartment E). The dynamics of the model can be described by the system of differential equations. These equations were exploited to define a physics loss function for training of Density-PINN, where the original S, E, I, R, and g were substituted with corresponding neural network approximations: $\tilde{S}$, $\tilde{E}$, $\tilde{I}$, $\tilde{R}$, and $\tilde{g}$, respectively. (B) Through a delayed stochastic simulation algorithm[5], we produced 50 time traces of the compartments S, E, I, and R with the transmission rate $\beta = 0.35$, recovery rate $\gamma = 0.25$, and the latent period distribution $g(t) \sim \Gamma(5, 1)$. These traces were then employed to define a data loss by quantifying the distance between the given traces and neural networks. (C) We trained Density-PINN by minimizing both physics and data losses. After training, Density-PINN yields accurate estimates of $g(t)$, including prediction intervals (black). As this result was presented for illustration of scalability of Density-PINN to an example other than cell-signaling pathway, we have made several assumptions helping the estimation of $g(t)$. Specifically, we assumed that both β and γ are known and time traces of all compartments S, E , I , and R are observable. While these assumptions resolve identifiability issues when we estimate $g(t)$, it may not practical in real-world applications. Therefore, relaxing these assumptions would be an important future avenue of research to explore.

Supplemental Tables

Table S1. Abbreviations for the promoters' name

Promoter	Abbrebation
wrba	wa
dps	ds
gadw	gw
iscR	iR
gadb	gb
cspA	cA
rpmE	rE
purM	pM
purT	pT
nrdH	nH
ldha	la
ahpC	aC
purT	pT
gada	ga
osmc	oc
dnaK	dK
ydiU	yU

Table S2. Selection of hyperparameters.

Part	Name	Numb. of Nodes	Activation functions[1]	Input
Data	Input	$Input\_dim\ (d)$	-	-
Encoder	Hidden_ 1	16	$ELU(\,)$	Input
	Hidden_ 2	16	$ELU(\,)$	Hidden_1
Latent	mean	$Latent\_dim\ (k = 4)$	-	Hidden _2
	logvar	$k = 4$	-	Hidden _2
Decoder $\boldsymbol{\omega}$	Hidden_3	16	$ELU(\,)$	[mean, logvar]
	Hidden_4	16	$ELU(\,)$	Hidden_3
	$\boldsymbol{\omega}$	$N\_comp\ (M = 80)$	$Softmax(\,)$	Hidden_4
Decoder $\boldsymbol{s}$	Hidden_5	16	$ELU(\,)$	[mean, logvar]
	Hidden_6	16	$ELU(\,)$	Hidden_5
	$\boldsymbol{s}$	$M = 80$	$(s_{max} - s_{min}) \times Sigmoid(\,) + s_{min}$	Hidden_6
Decoder λ_b, λ_d	Hidden_7	16	$ELU(\,)$	[mean, logvar]
	λ_b, λ_d	2	$ELU(\,) + 1$	Hidden_7
NN	Hidden_8	$M = 80$	$ELU(\,)$	$\boldsymbol{\omega}$
	$\boldsymbol{\omega}'$	$M = 80$	$ELU(\,) + 1$	Hidden_8

[1]The following activation functions $ELU(x)$, $Sigmoid(x)$ and $Softmax(x)$ were used:

$$ELU(x_j) = x_j \text{ if } x_j > 0, \qquad \exp(x_j) - 1 \text{ otherwise},$$

$$Sigmoid(x_j) = \frac{1}{1 + \exp(-x_j)},$$

$$Softmax(x_j) = \frac{e^{x_j}}{\sum_{k=1}^{J} e^{x_k}}.$$

Supplemental References

1. Bertsimas, D., and Mourtzinou, G. (1997). Transient laws of non-stationary queueing systems and their applications. Queueing Systems *25*, 115-155.
2. Kim, D.W., Hong, H., and Kim, J.K. (2022). Systematic inference identifies a major source of heterogeneity in cell signaling dynamics: The rate-limiting step number. Sci. Adv. *8*, eabl4598. 10.1126/sciadv.abl4598.
3. Kingma, D.P., and Ba, J. (2014). Adam: A method for stochastic optimization. arXiv preprint arXiv:1412.6980.
4. Kingma, D.P., and Welling, M. (2013). Auto-encoding variational bayes. arXiv preprint arXiv:1312.6114.
5. Cai, X. (2007). Exact stochastic simulation of coupled chemical reactions with delays. J Chem. Phys. *126*, 124108. 10.1063/1.2710253.
6. Gupta, C., Lopez, J.M., Azencott, R., Bennett, M.R., Josic, K., and Ott, W. (2014). Modeling delay in genetic networks: from delay birth-death processes to delay stochastic differential equations. J Chem. Phys. *140*, 204108. 10.1063/1.4878662.
7. Hartigan, J.A., and Hartigan, P.M. (1985). The dip test of unimodality. Ann.Stat., 70--84.
