# Supplementary material for: Density physics-informed neural networks reveal sources of cell heterogeneity in signal transduction
Source: Patterns (N Y). 2023 Dec 26;5(2):100899. doi: 10.1016/j.patter.2023.100899 (PMC10873160; doi:10.1016/j.patter.2023.100899)
Supplement: Document S2. Article plus supplemental information [file mmc2.pdf]

Patterns

Article

Density physics-informed neural networks reveal sources of cell heterogeneity in signal transduction

Graphical abstract

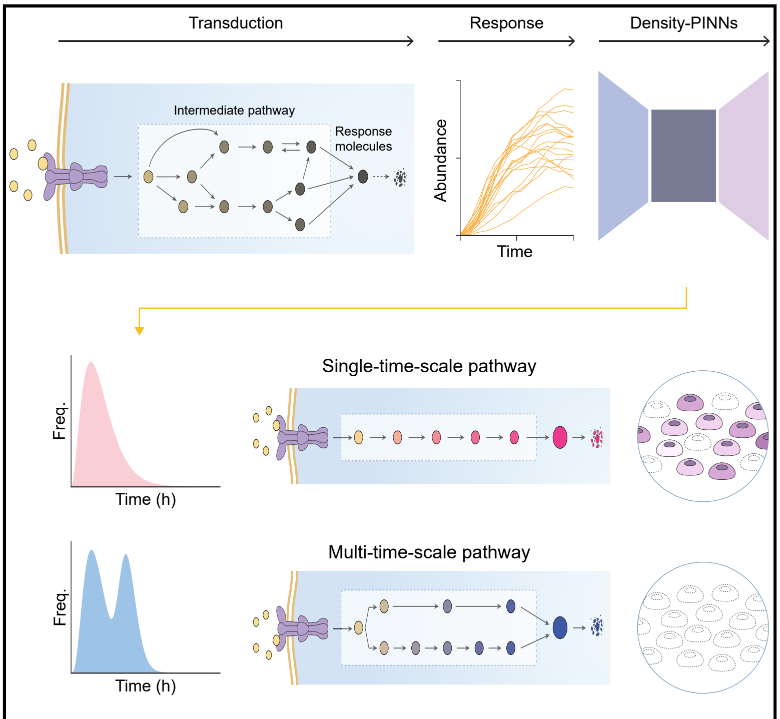

Highlights

- We build a method to infer the transduction-time distribution in a cell signaling pathway
- Pathways with longer response times show larger cell-to-cell heterogeneity in response
- Pathways with multiple timescales reduce the cell-to-cell heterogeneity in response
- Our method enables identifying effective signaling pathways for disease treatments

Authors

Hyeontae Jo, Hyukpyo Hong, Hyung Ju Hwang, Won Chang, Jae Kyoung Kim

Correspondence

jaekkim@kaist.ac.kr

In brief

Signaling pathways are essential for cell function. Their speed, precision, and structure can be inferred from the transduction time. Here, the authors develop a method, called density physics-informed neural networks (Density-PINNs), to estimate transduction-time distributions. By applying it to antibiotic response data, they find that pathways with a faster response or multiple timescales have reduced cell-to-cell heterogeneity in response. This finding suggests a way to identify signaling pathways that may provide more consistent cellular responses to disease treatments.

Jo et al., 2024, Patterns *5*, 100899
February 9, 2024 © 2023 The Author(s).
https://doi.org/10.1016/j.patter.2023.100899

CellPress

Patterns

CellPress
OPEN ACCESS

Article

Density physics-informed neural networks reveal sources of cell heterogeneity in signal transduction

Hyeontae Jo,[1,6] Hyukpyo Hong,[1,2,5,6] Hyung Ju Hwang,[3] Won Chang,[4] and Jae Kyoung Kim[1,2,7,*]
[1]Biomedical Mathematics Group, Pioneer Research Center for Mathematical and Computational Sciences, Institute for Basic Science, Daejeon 34126, Republic of Korea
[2]Department of Mathematical Sciences, KAIST, Daejeon 34141, Republic of Korea
[3]Department of Mathematics, Pohang University of Science and Technology, Pohang 37673, Republic of Korea
[4]Division of Statistics and Data Science, University of Cincinnati, Cincinnati, OH 45221, USA
[5]Present address: Department of Mathematics, University of Wisconsin – Madison, Madison, WI 53706, USA
[6]These authors contributed equally
[7]Lead contact
*Correspondence: jaekkim@kaist.ac.kr
https://doi.org/10.1016/j.patter.2023.100899

THE BIGGER PICTURE Understanding cellular signaling pathways is crucial because their dysregulation can lead to diseases and treatment resistance. For instance, if signaling pathways that respond to antibiotics or cancer therapeutics show a large heterogeneity in response between cells, some cells could survive the treatment, while others are killed by it. Valuable information about the signaling pathway, such as its speed, precision, and structure, can be inferred from the transduction time, the time it takes for a signal to travel from its initiation to its final response. Therefore, developing methods that can estimate the transduction-time distribution of a signaling pathway could enable the identification of sources of cellular heterogeneity and could ultimately help develop better treatment agents that can avoid or overcome heterogeneous cellular responses.

1 2 3 4 5 **Proof-of-Concept:** Data science output has been formulated, implemented, and tested for one domain/problem

SUMMARY

The transduction time between signal initiation and final response provides valuable information on the underlying signaling pathway, including its speed and precision. Furthermore, multi-modality in a transduction-time distribution indicates that the response is regulated by multiple pathways with different transduction speeds. Here, we developed a method called density physics-informed neural networks (Density-PINNs) to infer the transduction-time distribution from measurable final stress response time traces. We applied Density-PINNs to single-cell gene expression data from sixteen promoters regulated by unknown pathways in response to antibiotic stresses. We found that promoters with slower signaling initiation and transduction exhibit larger cell-to-cell heterogeneity in response intensity. However, this heterogeneity was greatly reduced when the response was regulated by slow and fast pathways together. This suggests a strategy for identifying effective signaling pathways for consistent cellular responses to disease treatments. Density-PINNs can also be applied to understand other time delay systems, including infectious diseases.

INTRODUCTION

Cells respond to signals from their extracellular environment through complex intracellular signaling pathways. While reliable signaling is necessary for proper cell function, the timing and strength of the response to the same extracellular signal can vary significantly, even in genetically identical cell populations.[1–9] This cell-to-cell heterogeneity leads to the emergence

This is an open access article under the CC BY license (http://creativecommons.org/licenses/by/4.0/).

of abnormal cells, which can cause disease. Furthermore, heterogeneity can lead to incomplete killing of target cancer cells[10] and the emergence of persister cells,[11] which are major obstacles in effectively treating cancer.

Previous studies have focused on indirect sources of cell-to-cell heterogeneity in signaling responses,[12–15] such as cell-cycle phase[16] and RNA polymerase level.[17] On the other hand, direct sources, i.e., the signaling pathways themselves, have been investigated in a limited number of studies when comprehensive information about the pathways is known.[18–20] For example, Granados et al. modified known signaling pathways leading to Hog1 expression and found that yeast shows low cell-to-cell heterogeneity in response to osmotic stress when the response is regulated by multiple pathways with both slow and fast signaling transduction speeds.[18] Chepyala et al. developed a mathematical model for a known regulatory pathway in *Caenorhabditis elegans* and investigated the role of the pathways in controlling the heterogeneity of distal tip cell migration timing.[20] However, comprehensive information about signaling pathways is rarely known except for these limited cases, rendering it challenging to identify the sources of the cell-to-cell heterogeneity within the signaling pathways themselves.

To overcome this lack of information about signaling pathways, one promising solution is to develop a model by replacing an unknown pathway with a single random time delay.[21–28] This random time delay describes the time it takes between the signal activation and the production of response molecules through the unknown pathway, also known as signal transduction time (Figure 1A). The shape of the transduction-time distribution provides information about the underlying signaling pathway. For instance, the low mean and variance of the distribution indicate that the underlying pathway is fast and precise, respectively (Figure 1B). Furthermore, the number of modes in the distribution provides information about the structure of the underlying pathway.[25] A unimodal transduction-time distribution emerges when the response is regulated by a single-timescale pathway (e.g., an irreversible chain and a reversible cascade) (Figure 1C, top). On the other hand, a transduction time is multi-modal when the response is regulated by multiple pathways with different transduction speeds, i.e., a multi-timescale pathway (e.g., a cross-talk and feedforward network) (Figure 1C, bottom). This indicates that inferring the shape of the transduction-time distribution can provide valuable information about the characteristics of the underlying signaling pathway.

Kim et al. developed a parametric Bayesian inference method that infers transduction-time distributions, revealing the rate-limiting steps in a signaling pathway.[21] Through this approach, this study found that as the number of rate-limiting steps increases, so does cell-to-cell heterogeneity in response to antibiotic stress. However, this method only works if the underlying signaling pathway is a single-timescale pathway, resulting in the transduction-time distribution following a gamma distribution (Figure 1C, top). Similarly, other inference methods have limitations,[21–24] as they are only applicable when sufficient information about the signaling pathway is available to specify the type of transduction-time distribution.

In this article, we describe a method we have developed, density physics-informed neural networks (Density-PINNs), which infer the shape of transduction-time distributions in signaling pathways only from time traces of the final stress response. Specifically, we modified PINNs[29] to incorporate physics-based knowledge of a signaling process with an arbitrary transduction-time distribution into the training of neural networks (NNs). We applied Density-PINNs to single-cell gene expression time traces from 16 promoters in response to the antibiotic stresses tetracycline (TET) and trimethoprim (TMP). This allowed us to uncover key features of unknown signaling pathways regulating these promoters, including the speed of signal initiation and transduction, transduction-time precision, and whether the promoter is regulated by single- or multi-timescale pathways. Importantly, we found that promoters with longer signaling initiation and transduction time (i.e., longer response time) exhibit larger cell-to-cell heterogeneity in response intensity. However, this heterogeneity is greatly reduced when the response is regulated by multi-timescale pathways (Figure 1C, bottom). This finding suggests that targeting pathways with shorter response times or involving multi-timescale pathways can enhance the consistency of cellular responses and decrease unresponsive cells, which is critical for the development of anticancer drugs. Density-PINNs provide an effective method to gain critical information about cell signaling pathways only from their response time traces.

RESULTS

Cellular processes with hidden reactions can be described with a delayed model

Intracellular signaling pathways, activated by extracellular stimuli, can be described by a stochastic delayed birth-death process.[30–33] In this model, the signal is activated at a rate of λ_b, and then it is transduced via signaling pathways and triggers the final response after a distributed time delay $g(t)$, and the final response molecules decay at a rate of λ_d (Figure 1D). In this way, unobserved complex intermediate steps can be simply described with a delay distribution $g(t)$. The transduction-time distribution $g(t)$ is unimodal or multi-modal depending on whether the underlying signaling pathways have single or multiple timescales (Figure 1C). The mean time trace of this stochastic process $y(t)$ can be described by the following equation[21] (see Note S1 for details):

$$\frac{dy}{dt} = \lambda_b \int_0^t g(s)ds - \lambda_d y(t). \qquad \text{(Equation 1)}$$

Density-PINNs: PINN-based estimation method for transduction-time distribution

As this formula provides a connection between the underlying transduction-time distribution $g(t)$ and the final response $y(t)$, it can be used to estimate the $g(t)$ from the $y(t)$. One promising approach for this purpose is to use PINNs, which are deep learning methods that integrate data and governing equations to estimate parameters. However, conventional PINNs can estimate parameter values rather than a probability distribution.[34,35] To address this problem, we propose Density-PINNs that yield distribution $\tilde{g}(t)$ as the estimates of $g(t)$ (see Note S7 for a step-by-step manual). Specifically, we used M Rayleigh distributions with different modes and widths as building blocks to construct the arbitrary distributions: $\tilde{g}(t) := \sum_{j=1}^{M} \omega_j K(t; c_j, s_j)$, where c_j and s_j determine the mode and widths of Rayleigh distribution $K(t; c_j, s_j)$. Thus, by estimating the parameters

CellPress
OPEN ACCESS

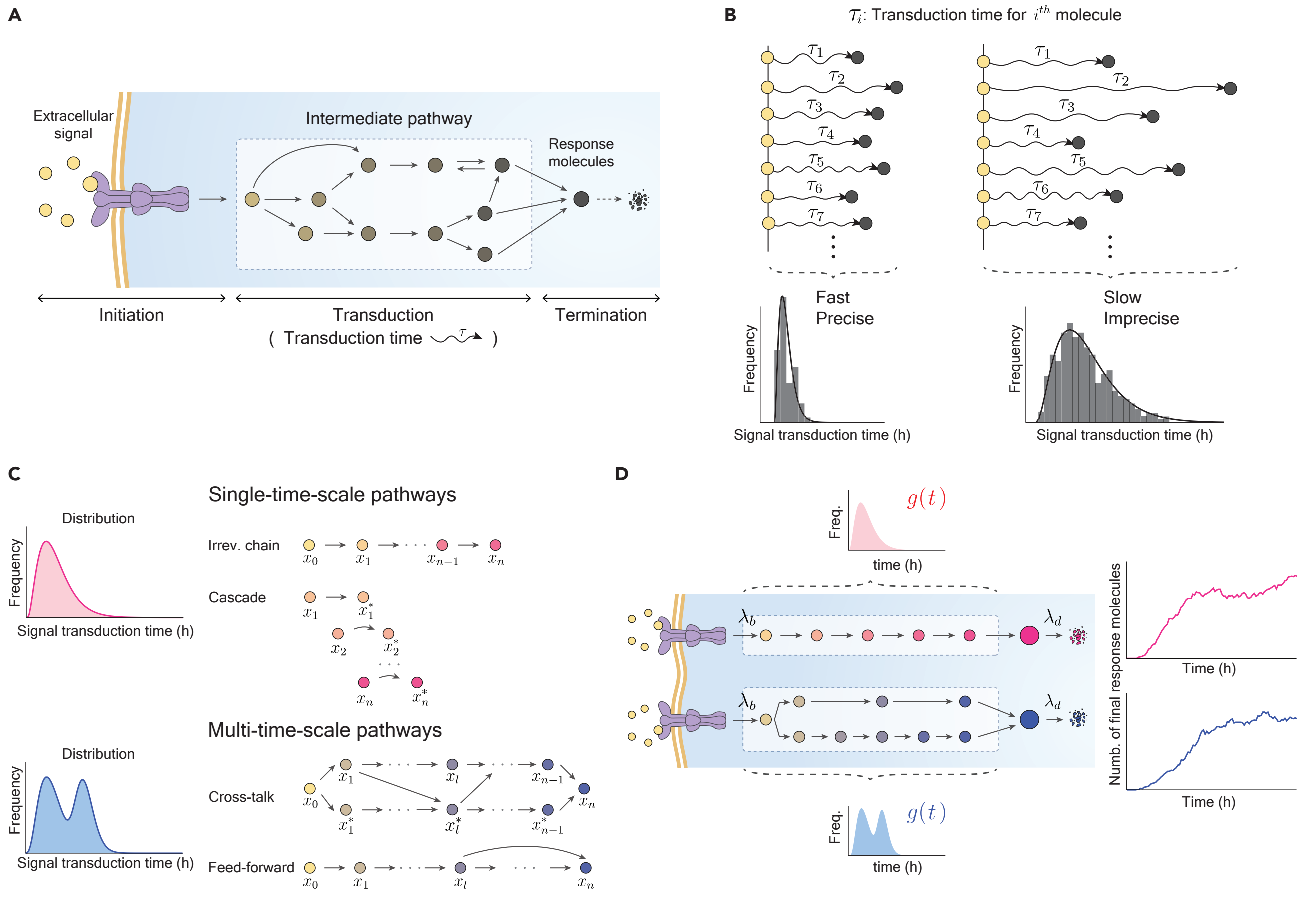

Figure 1. The distribution of signal transduction time provides rich information about the underlying signaling pathways

(A) The signal transduction time refers to the time it takes between signal initiation and production of the final response molecules through intermediate pathways.

(B) If the signal transduction is precise and fast (or imprecise and slow), then the distribution of the transduction time (τ_i) becomes narrow (or wide) and has a small (or large) mean.

(C) Single- and multi-timescale pathways have different numbers of modes of the transduction-time distribution.

(D) The signaling pathways are modeled using a stochastic delayed birth-death process. The signal is activated at a rate of λ_b and transduced via signaling pathways. The response molecules are produced after the delay, whose distribution is $g(t)$, and they decay at a rate of λ_d. Although single- and multi-timescale pathways have different transduction-time distributions (unimodal and multimodal), time traces of the final product are indistinguishable.

(ω_j, c_j, s_j) of $\tilde{g}(t)$, Density-PINNs can estimate transduction-time distribution.

To perform efficient parameter estimation, we used evenly spaced shift parameters c_j within the time domain of the observed time series data $y(t)$. Then, the remaining scale parameters s_j and weights ω_j were estimated through an artificial NN based on a variational autoencoder (VAE) (Figure 2A, VAE).[36,37] Specifically, the artificial NN maps $y(t)$ to a low-dimensional latent variable $\boldsymbol{z}$ and generates distributions of s_j and ω_j from $\boldsymbol{z}$ (see methods for details). From the distributions of s_j, ω_j, and predetermined c_j, a probability distribution of $\tilde{g}(t)$ can be constructed (Figure 2A, output).

To train $\tilde{g}(t)$ so that it approximates the true transduction-time distribution, $g(t)$, we need to define a loss function. Because $g(t)$ is unobservable, we cannot directly measure the difference between $g(t)$ and $\tilde{g}(t)$. Instead, we indirectly quantified this difference by comparing the observed $y(t)$ to the reconstructed $\tilde{y}(t)$ obtained with $\tilde{g}(t)$ (Figure 2A, output; see Note S2 for details). To make sure that $\tilde{y}(t)$ approximates $y(t)$ and satisfies the governing equation (Equation 1), we used a data loss $\|y - \tilde{y}\|$ and physics loss $\|\frac{d\tilde{y}}{dt} - (\lambda_b \int_0^t \tilde{g}(\tau)d\tau - \lambda_d\tilde{y})\|$ (Figure 2B). Since the physics loss contains λ_b and λ_d, they were also estimated through a separate artificial NN (Figure 2A, output). Furthermore, we included a regularization loss for scale parameters of $K(t; c_j, s_j)$, $\|s\|$, and a typical regularization loss of VAE, Kullback-Leibler (KL) divergence $D_{\mathrm{KL}}(\boldsymbol{z}\|\mathrm{N}(\boldsymbol{0},\boldsymbol{I}))$. $\|s\|$ prevent s_j from having too small or too large values (see Note S3 for details), and $D_{\mathrm{KL}}(\boldsymbol{z}\|\mathrm{N}(\boldsymbol{0},\boldsymbol{I}))$ ensures informative representation of data with latent variables.

For multiple input time traces $\{y_1(t), y_2(t), \ldots, y_\mathrm{N}(t)\}$, the average of the total loss function (Figure 2B) of each time trace was minimized for the training of Density-PINNs (Figure 3A, dashed box). Then, we estimated the distribution of $g(t)$, λ_b, and λ_d using the mean time trace $\bar{y}(t)$ (Figure 3A).

Density-PINNs accurately estimate transduction-time distributions

We tested whether Density-PINNs could estimate the three distinct transduction-time distributions: unimodal, weakly

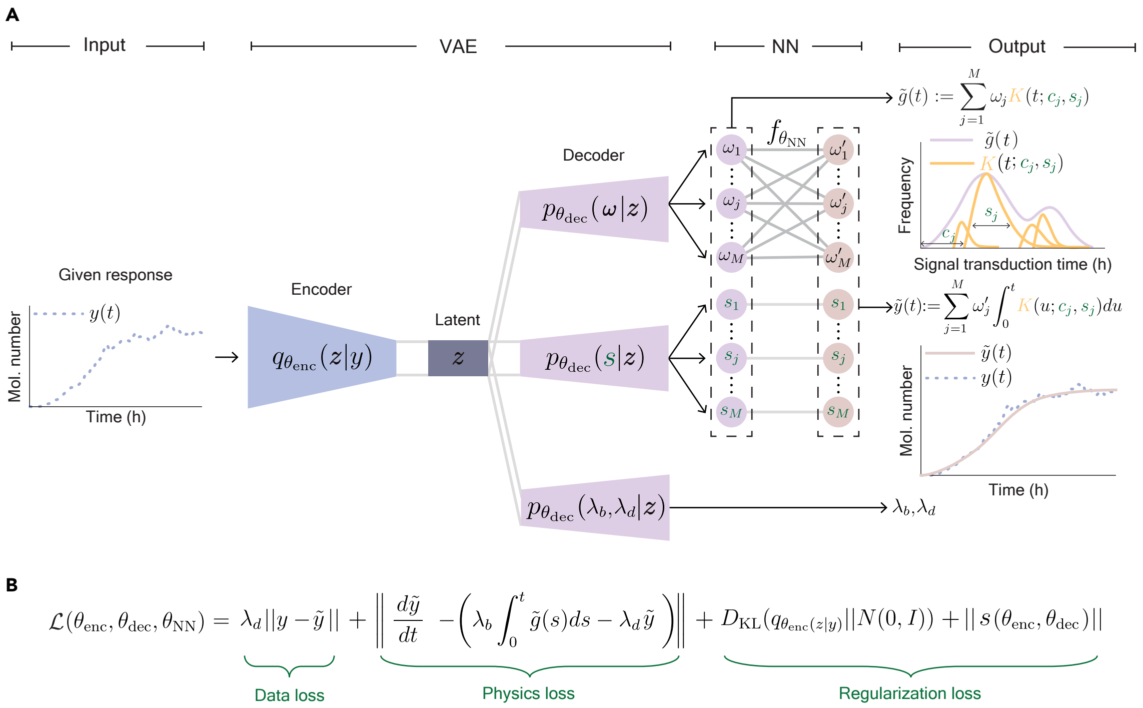

Figure 2. Schematic architecture of the framework of Density-PINNs for inferring a delay distribution

(A) From the input response $y(t)$ processed through a variational autoencoder (VAE), a neural network (NN) provides the estimated transduction-time distribution $\tilde{g}(t)$, as well as the activation rate λ_b, the decay rate λ_d, and the reconstructed response $\tilde{y}(t)$. Specifically, in the VAE, the encoder $q_{\theta_{\text{enc}}}$, governed by the trainable parameters θ_{enc}, maps $y(t)$ to a latent variable $\boldsymbol{z}$ in the latent space Z, and the decoder $p_{\theta_{\text{dec}}}$, governed by the trainable parameters θ_{dec}, maps the latent variable $\boldsymbol{z}$ to a weight ω, scale parameter s, λ_b, and λ_d. The NN $f_{\theta_{\text{NN}}}$, governed by trainable parameters θ_{NN}, maps ω to the other weight ω'. ω',ω, and s are used to compute $\tilde{g}(t)$ and $\tilde{y}(t)$ as $\sum_{i=1}^{M} \omega_i K(t; c_i, s_i)$ and $\sum_{i=1}^{M} \omega'_i \int_0^t K(u; c_i, s_i) du$, respectively, where $K(u; c_i, s_i)$ is a shifted Rayleigh density with the shift parameter c_i and the scale parameter s_i.

(B) The total loss function is composed of the data loss, physics loss, and regularization loss. The data loss quantifies the distance between $y(t)$ and $\tilde{y}(t)$. The physics loss quantifies how well $\tilde{g}(t)$ and $\tilde{y}(t)$ fit the governing equation (Equation 1). The regularization loss simultaneously prevents $\boldsymbol{s}$ from becoming too small or too large and ensures effective representation of the data by the latent variables $\boldsymbol{z}$.

bimodal, and strongly bimodal distributions (Figure 3B, input). We first generated 50 traces with each transduction-time distribution using a delayed stochastic simulation algorithm (see Note S5 for details).[38] Then, we used these traces to estimate a transduction-time distribution $\tilde{g}(t)$, an activation rate λ_b, and a decay rate λ_d. Although the generated traces were nearly indistinguishable, our method successfully estimated the true transduction-time distributions for all three transduction-time distributions (Figure 3B, output). In particular, the true transduction-time distributions are fully contained within the quantified prediction intervals (Figure 3B, output, shaded region; see methods for details). Moreover, the transduction-time distributions were accurately estimated even when the underlying transduction-time distribution had three modes or a flat peak (Figure S2) and when time traces contained multiplicative measurement noise (Figure S3).

We repeated this estimation 100 times by generating 100 different datasets, each containing 50 traces. Our method consistently provided accurate estimates of λ_b (Figure 3C) and λ_d (Figure 3D), the small distance between the true $g(t)$ and the estimated $\tilde{g}(t)$ as quantified by KL divergence (Figure 3E), and accurate estimates of bimodality as quantified by a dip statistic (Figure 3F; see Note S6 for details). The accuracy and precision of the estimations improve as the number of time traces used for the estimations increases (Figures 3C–3F).

When the decay mainly occurs via growth-induced dilution, the decay rate can be replaced with a dilution rate whose value can be estimated by measuring single-cell growth trajectories obtained with time-lapse microscopy. Thus, we tested our method when the decay rate λ_d was fixed to its true value. In this case, the estimations for the transduction-time distribution $g(t)$ and the activation rate λ_b became more accurate and precise (Figure S4).

Multi-timescale pathways reduce the cell-to-cell heterogeneity in response

We applied our method to the previously measured single-cell time-lapse yellow fluorescent protein (YFP) expression data from 16 promoters in response to two antibiotic stresses, TET and TMP, in *Escherichia coli* (*E. coli*) populations (Figure 4A).[2] The response time traces showed significant cell-to-cell heterogeneity. In particular, the final stress intensity is highly variable, which we quantified using a coefficient of variation (CV) calculated at the final observation point, referred to as the population CV of y. The population CV of y showed large differences over the promoters (from 0.18 to 0.66; Figure 4A), even for the same antibiotic stress. However, it was unclear which properties of the signaling pathways affect the cell-to-cell heterogeneity in response due to the limited information available on the signaling pathways for each promoter.

CellPress
OPEN ACCESS

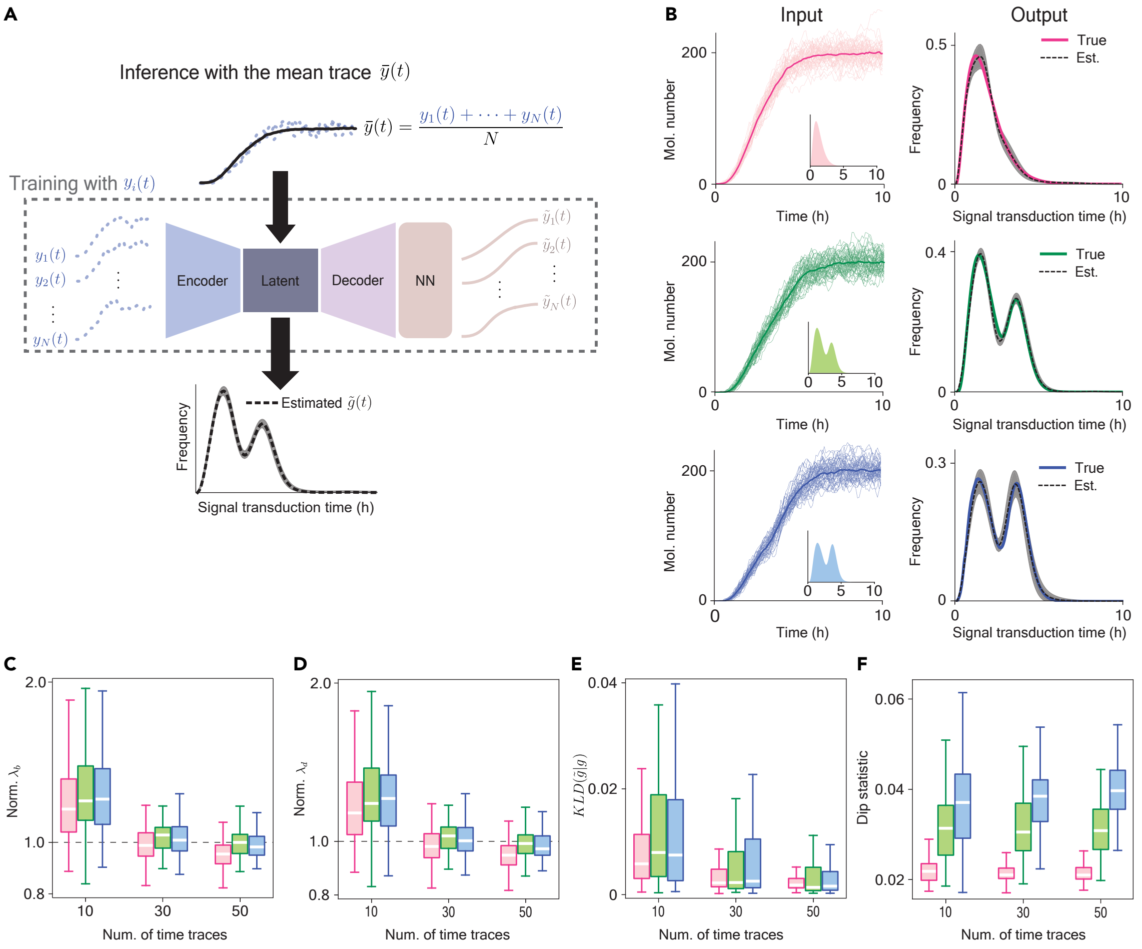

Figure 3. Density-PINNs accurately estimate underlying transduction-time distributions with various shapes

(A) We trained Density-PINNs with N individual response time traces so that the average of the total losses from N time traces is minimized. Using the trained model, a transduction-time distribution $\tilde{g}(t)$ as well as λ_b and λ_d were inferred using the mean of the N time traces, $\bar{y}(t)$, as an input.

(B) Density-PINNs accurately infer the underlying transduction-time distributions (output) from simulated 50 time traces (input) when the transduction-time distribution is unimodal (red), weakly bimodal (green), or strongly bimodal (blue) (input; inset). The shaded region represents the prediction intervals of the estimated transduction-time distribution (see methods for details). Here, data were sampled every 0.5 h.

(C–F) As more time traces were used for the inference, the estimation became more accurate: estimations of λ_b and λ_d became more accurate and precise (C and D), the KL divergence between the underlying and reconstructed transduction-time distributions decreased (E), and the dip statistic, which increases as the bimodality increases, became more clearly distinguished among the unimodal (red), weakly bimodal (green), and strongly bimodal (blue) distributions (F). Here, boxplots indicate the first and third quartiles, and whiskers extend from each box to the farthest data point lying within 1.5 times the inter-quartile range.

To obtain information about the signaling pathways, we estimated the signal initiation time λ_b^{-1}, the time it takes to begin signal transduction in response to antibiotics, and the transduction-time distribution $g(t)$ of each promoter using Density-PINNs. Throughout the estimation, the dilution rate, directly estimated from the experimentally measured cell growth rate,[2] was used as the decay rate λ_d because dilution is the main driver of the decay of YFP.[39] Our inference results provided valuable information on the unknown signaling pathways regulating these promoters. Specifically, out of 16 promoters, 12 promoters exhibited unimodal transduction-time distributions (Figure 4B, red), while the other four promoters exhibited multi-modal transduction-time distributions (Figure 4B, blue). This indicates that the 12 promoters are regulated by single-timescale pathways (Figure 1C, top), while the other four promoters are regulated by multi-timescale pathways (Figure 1C, bottom). Furthermore, the mean μ_g, the standard deviation σ_g, and the CV (=σ_g/μ_g) of each transduction-time distribution $g(t)$ across the promoters reveal the speed and precision of the signal transduction time (Figure 4C).

We next investigated the relationship between these quantified characteristics of signaling pathways and the cell-to-cell heterogeneity (i.e., population CV of y). Interestingly, the cell-to-cell heterogeneities in response to promoters regulated by single-timescale pathways were higher compared to those regulated by multi-timescale pathways (Figure 4D). However, none of the other characteristics, such as the means, standard deviations, and CVs of transduction-time distributions, were significantly correlated with the cell-to-cell heterogeneity in response ($p > 0.05$; Figure 4E) except for the signal initiation time λ_b^{-1} in multi-timescale pathways (Figure 4E, bottom right). In particular, it was unexpected that a large variation in transduction-time

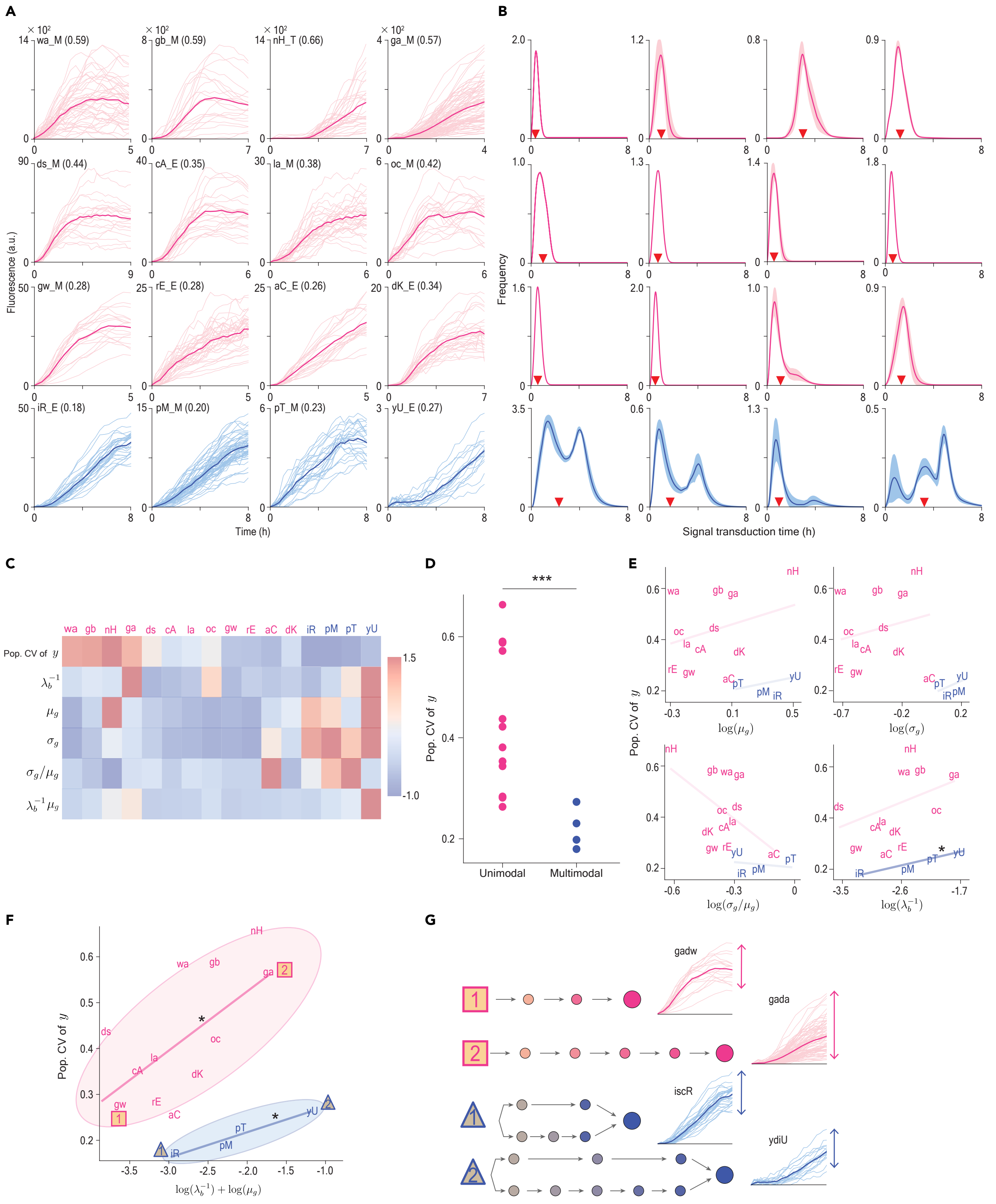

Figure 4. Multi-timescale pathway leads to low cell-to-cell heterogeneity in response to antibiotic stresses

(A) Response time traces of 16 promoters to the antibiotic stresses, TET (E) and TMP (M), were measured by time-lapse fluorescence microscopy.[2] The thin lines represent single-cell time traces, while the thick lines indicate the mean time traces. The numbers in parentheses are the coefficients of variation (CVs) of the responses at the final observation point. See Table S1 for the abbreviations of the promoters.

(legend continued on next page)

CellPress
OPEN ACCESS

distribution (i.e., a highly variable timing of response) did not lead to a large cell-to-cell heterogeneity in final response intensity.

Unlike the signal initiation time λ_b^{-1} and the mean of transduction time μ_g, interestingly, their sum, i.e., the response time, is significantly correlated with the cell-to-cell heterogeneity in both single- and multi-timescale pathways (Figure 4F). These results align with previous studies that have revealed that positive and negative autoregulation increases and decreases, respectively, the response time and cell-to-cell heterogeneity at the protein level.[40–42] Our observations are also consistent with a previous study that showed the positive correlation between the cell-to-cell heterogeneity and the number of rate-limiting steps in signal transduction pathways, which is positively correlated with the response time.[21]

Moreover, we found that the positive relationship was stronger in the single-timescale pathway group compared with the multi-timescale pathway group. In particular, when the response time was short (e.g., for *gadw* and *iscR* promoters), the cell-to-cell heterogeneity in response to antibiotic stress was small (Figure 4F and 4G, rectangle and triangle 1). When the response time was long (e.g., for *gada* and *ydiU* promoters), the cell-to-cell heterogeneity was large if the response was regulated by a single-timescale pathway (Figures 4F and 4G, rectangle 2), while the heterogeneity was still relatively small if the response was regulated by a multi-timescale pathway (Figures 4F and 4G, triangle 2). Interestingly, this result is consistent with previous findings that multi-timescale pathways reduce cell-to-cell heterogeneity in cell volume recovery,[18] which was concluded from intensive experimental work using mutant yeast cells.

DISCUSSION

We developed Density-PINNs that accurately infer parameter values and transduction-time distribution of a stochastic process with time delay (Figures 2 and 3). We applied Density-PINNs to single-cell time-lapse fluorescent protein expression data in response to two antibiotic stresses, TET and TMP (Figures 4A–4C). This uncovered key properties of the signaling pathways leading to the cell-to-cell heterogeneity in stress response: an increase in heterogeneity with longer response time (Figure 4F) and a decrease in heterogeneity when triggered by multi-timescale pathways (Figure 4G). These results highlight the importance of response time and pathways, which can be inferred with our method, in identifying effective target molecules for drug development (Figure 5). Our findings also enable a systematic understanding of the heterogeneity of treatment effects, which is a major challenge for precision medicine.[43]

We illustrated the application of Density-PINNs with a focus on cases where the final response increases and then saturates, which can be described by Equation 1. For more complex dynamics, such as adaptation or oscillation profiles, the mean formula of the stochastic delayed birth-death process with feedback regulation, derived in our previous work,[21] can be utilized instead of Equation 1. To further extend to other dynamics such as switch-like (i.e., ultrasensitive) response,[44,45] the mean formula for the stochastic process that describes the dynamics needs to be derived. Then, by simply adjusting the physics loss term based on the mean formula,[46,47] Density-PINNs can be applied to analyze signaling pathways with a wider range of dynamics.

Recently, PINNs have emerged as a powerful tool for inferring parameter values of differential equations because they incorporate prior physical knowledge within NNs while accurately fitting data. In this study, we proposed Density-PINNs that estimate a probability distribution as well as parameter values. Density-PINNs produce outputs that naturally conform to the properties of probability distributions by using the sum of probability density functions as done in a previous study.[48] In this way, additional loss terms to impose constraints on the probability distributions, causing additional computational costs, were not needed, unlike in previous approaches.[34,35] To improve computational efficiency further, we chose the Rayleigh distribution as the kernel density since it satisfies the vanishing condition at time $t \leq 0$ and allows direct calculation of definite integration of the estimated distribution in the physics loss without numerical integration, unlike the negative binomial, which was in a previous study.[48]

This study has several limitations. First, the decay of proteins was described by a first-order reaction in Equation 1, which does not account for the change of decay rate in time during the cell cycle. An explicit description of the cell cycle using, for example, binomial partitioning of the proteins at cell division would allow us to apply the current method to a more sophisticated model.[49] Second, while our method successfully estimated the transduction-time distribution for birth-death processes and an infectious disease model with delay (Figures 3 and S6), recent studies have shown that the original PINNs do not work for rapidly changing systems.[50,51] Therefore, a new learning strategy for Density-PINNs would be needed to improve their performance on such non-trivial systems.

PINNs have demonstrated their versatility in solving a wide variety of problems across different fields of science and engineering.[46] PINNs were originally used to solve deterministic differential equations[29,34,52] and their application was extended to optimize engineering designs,[53] solve inverse problems,[54,55] and stochastic

(B) Estimated transduction-time distributions from the response time traces in (A). The estimated transduction-time distributions of twelve and the other four populations exhibit unimodality (red) and bimodality (blue), respectively. Red triangles represent the mean transduction times.

(C) The characteristics of signaling pathways for each promoter quantified by Density-PINNs: the initiation time λ_b^{-1}, the mean μ_g, the variance σ_g^2, and the CV (=σ_g/μ_g) of transduction-time distribution. Additionally, we obtained the response time (=$\lambda_b^{-1}\mu_g$) (see methods for details). Here, each quantity was standardized so that the mean and variance among the promoters are zero and one, respectively.

(D) Population CVs of y were generally higher in the promoters with unimodal transduction-time distributions compared to those in the promoters with bimodal transduction-time distributions. Two-sided t test was used for statistical test, p = 0.0003 (***p < 0.001).

(E) None of μ_g, σ_g, σ_g/μ_g, and λ_b^{-1} were significantly correlated with population CVs of y, except for λ_b^{-1} of multi-timescale pathways (right bottom, blue). p values were calculated by the Pearson correlation test: p = 0.164 (0.699), 0.605 (0.525), 0.055 (0.791), and 0.063 (0.035) for μ_g, σ_g, σ_g/μ_g, and λ_b^{-1} in single (multi)-timescale pathways, respectively.

(F) On the other hand, the response time $\log \lambda_b^{-1} + \log \mu_g$ was significantly correlated with population CVs of y with p = 0.023 and 0.024 in single- and multi-timescale pathways. This correlation was smaller in multi-timescale pathways.

(G) Promoters with short response times (e.g., *gadw* and *iscR*) or involving multi-timescale pathways (e.g., *iscR and ydiU*) show a low level of heterogeneity.

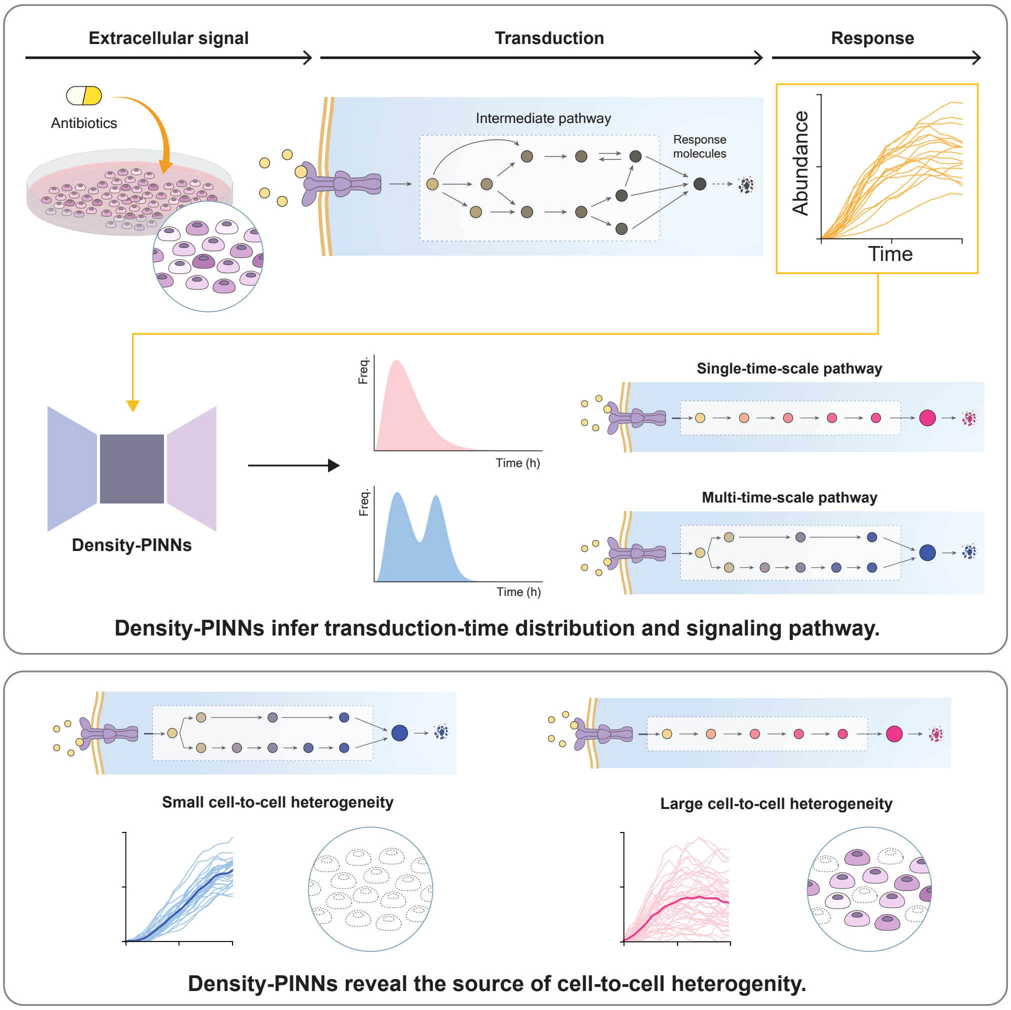

Figure 5. Summary figure

Density-PINNs reveal the source of cell-to-cell heterogeneity by inferring transduction-time distributions. (Top) When *E. coli* cells are exposed to antibiotic stress, responding proteins are produced via signaling pathways. From the time trace of the accumulated responding proteins, Density-PINNs infer the distribution of signaling transduction time, whose shape informs the number of pathways with different timescales. (Bottom) This revealed that the presence of signaling pathways with different timescales greatly reduces the cell-to-cell heterogeneity in response to antibiotics.

differential equations.[56–58] In this study, we pioneer the use of PINNs in the analysis of non-Markovian systems with distributed time delays, which can be applied to a variety of fields. For example, Density-PINNs can be used to infer the distribution of time delays due to the latent period of COVID-19[59] (Figure S6). Additionally, Density-PINNs can be used to calculate a lower bound of entropy production rate of a system by inferring a residence time distribution in each state of a non-Markovian model.[60] This is particularly important, as the entropy production rate quantifies the extent to which a cell is consuming energy in order to resist environmental changes.[61]

EXPERIMENTAL PROCEDURES

Resource availability

Lead contact

Requests for further information should be directed to and will be fulfilled by the lead contact, Jae Kyoung Kim (jaekkim@kaist.ac.kr).

Materials availability

This study did not generate new unique reagents.

Data and code availability

All original codes have been deposited at GitHub under https://github.com/mathbiomed/density-pinns and at Zenodo under https://doi.org/10.5281/zenodo.10108680[62] and are publicly available as of the date of publication. The experimental data used in this article can be accessed at Kim et al.[21]

Methods

Formulation of Density-PINN architecture

$Y = \{\boldsymbol{y}_1, \boldsymbol{y}_2, \ldots, \boldsymbol{y}_N\}$ is the set of N time traces whose mean and standard deviation are $\mu_Y(t)$ and $\sigma_Y(t)$, respectively. The standardized Y by replacing $y_i(t)$ with $(y_i(t) - \mu_Y(t))/\sigma_Y(t)$ was used as input of the encoder of the VAE. The encoder then mapped each standardized $\boldsymbol{y}_i$ to two parameters, $\boldsymbol{\mu}_i$ and $\boldsymbol{\sigma}_i^2$, used to parametrize the lower-dimensional latent variable $\boldsymbol{z}_i = \boldsymbol{\mu}_i + \boldsymbol{\sigma}_i \times \boldsymbol{\varepsilon}$ where $\boldsymbol{\varepsilon} \sim \mathrm{N}(\boldsymbol{0}, \boldsymbol{I})$:

$$q_{\theta_{enc}}(\boldsymbol{z}_i|\boldsymbol{y}_i) \sim \mathrm{N}\left(\boldsymbol{\mu}_i, \mathrm{diag}\left(\boldsymbol{\sigma}_i^2\right)\right),$$

where $\boldsymbol{\sigma}_i^2$ is the element-wise square of $\boldsymbol{\sigma}_i$. The dimension of $\boldsymbol{z}_i$ (k) is 4 and is much smaller than the dimension of $\boldsymbol{y}_i$ (d) used in this study. Thus, the encoder

consists of one input layer of size d, two hidden layers of size 16, and two output layers of size $k = 4$ for $\boldsymbol{\mu}_i$ and $\log \boldsymbol{\sigma}_i^2$. The goal of the encoder is to train the parameter θ_{enc} such that $q_{\theta_{\text{enc}}}(\boldsymbol{z}|\boldsymbol{y})$ is as close as possible to the true posterior distribution.

The decoder of the VAE $p_{\theta_{\text{dec}}}$ with the trainable parameter θ_{dec} consists of three fully connected NNs. The first two networks have the same architecture with two hidden layers of size 16, and the last network has one hidden layer of size 16. The three NNs transform the latent variable $\boldsymbol{z}_i$ to the weights $\boldsymbol{\omega}_i$, the scale parameter $\boldsymbol{s}_i$, and the activation and decay rates $(\lambda_{b,i}, \lambda_{d,i})$ to obtain the estimated transduction-time distribution $\tilde{g}_i(t)$ with predefined shift parameters $\{c_1, \ldots, c_M\}$ as follows:

$$\tilde{g}_i(t) = \sum_{j=1}^{M} \omega_i(c_j) K(t; c_j, s_i(c_j)),$$

where $K(t; c_j, s_i(c_j))$ is a shifted Rayleigh density with the scale parameters $s_i(c_j)$ and the shift parameters c_j weighted by $\omega_i(c_j)$ (Figure 2A, bottom). That is, $K(t; c_j, s_i(c_j)) = \frac{t - c_j}{s_i^2} \exp \frac{-(t - c_j)^2}{2s_i^2}$ for $t \geq c_j$ and $K(t; c_j, s_i(c_j)) = 0$ otherwise. We chose the Rayleigh distribution rather than negative binomial[36] or Gaussian distribution,[37] used in previous studies, as it has positive support. Moreover, this choice allowed the definite integration of $\tilde{g}_i(t)$ in the physics loss, which reduces the computational cost and numerical error of numerical integration. See Table S2 for the hyperparameters in Density-PINNs.

The VAE whose output is $\tilde{g}_i(t)$ cannot be directly trained because the true transduction-time distribution $g_i(t)$ is unobservable. Thus, we used $\tilde{y}_i(t)$ reconstructed from $\tilde{g}_i(t)$ and compared it with the true $\boldsymbol{y}_i$ (Figure S1). To do this, we employed an NN, $f_{\theta_{\text{NN}}}$, which has trainable parameters θ_{NN}. The NN is composed of an input layer, a hidden layer, and an output layer, each sized equally to the length of $\boldsymbol{\omega}_i$. Thus, the NN mapped weights $\boldsymbol{\omega}_i$ to new weights $\boldsymbol{\omega}_i'$ for reconstructing $\tilde{y}_i(t)$ as follows:

$$\tilde{y}_i(t) = \sum_{j=1}^{M} \omega_i'(c_j) \int_0^t K(u; c_j, s_i(c_j)) du.$$

Training of Density-PINNs

For the training, we used the Adam optimizer,[63] and to prevent overfitting issues, we applied an early stopping criterion[64] (see Note S4 for details). Density-PINNs were trained by minimizing the total loss function:

$$\mathcal{L}(\theta) = \mathcal{L}_d(\theta) + \mathcal{L}_p(\theta) + \mathcal{L}_r(\theta) \quad \text{(Equation 2)}$$

where $\theta = (\theta_{\text{enc}}, \theta_{\text{dec}}, \theta_{\text{NN}})$, $\mathcal{L}_d$ is a data loss, $\mathcal{L}_p$ is a physics loss, and $\mathcal{L}_r$ is a regularization loss. The data loss function is defined as

$$\mathcal{L}_d(\theta) = \frac{1}{\text{N}d} \sum_{i=1}^{\text{N}} \sum_{j=1}^{d} \left| \tilde{y}_i(t_j) - y_i(t_j) \right|,$$

where $\{t_1, \ldots, t_d\}$ is the set of observed time points of $y_i(t)$. The physics loss is evaluated the set of collocation points, $\{t_1^c, \ldots, t_C^c\}$, evenly spaced in the time domain $[0, T]$:

$$\mathcal{L}_p(\theta) = \frac{1}{\text{N}C} \sum_{i=1}^{\text{N}} \sum_{j=1}^{C} \left| \frac{d\tilde{y}_i}{dt}\left(t_j^c\right) - \left(\lambda_b \int_0^{t_j^c} \tilde{g}_i(s) ds - \lambda_d \tilde{y}_i\left(t_j^c\right) \right) \right|,$$

where the derivative of $\tilde{y}_i$ with respect to time t is computed using automatic differentiation.[65] The regularization loss is computed as follows:

$$\mathcal{L}_r(\theta) = \|s(\theta_{\text{enc}}, \theta_{\text{dec}})\| + D_{\text{KL}}\left(q_{\theta_{\text{enc}}}(\boldsymbol{z}|\boldsymbol{y}) \middle\| \text{N}(\boldsymbol{0}, \boldsymbol{I})\right).$$

The first term is given by

$$\|s(\theta_{\text{enc}}, \theta_{\text{dec}})\| = \frac{1}{\text{N}d} \sum_{i=1}^{\text{N}} \sum_{j=1}^{d} \left| s_i(t_j) - (\beta_i(t_j) s_{\min} + (1 - \beta_i(t_j)) s_{\max}) \right|,$$

where $\beta_i(t_j) = \frac{\frac{d\langle \boldsymbol{y}_i \rangle_+}{dt}(t_j) - \min \frac{d\langle \boldsymbol{y}_i \rangle_+}{dt}(t)}{\max \frac{d\langle \boldsymbol{y}_i \rangle_+}{dt}(t) - \min \frac{d\langle \boldsymbol{y}_i \rangle_+}{dt}(t)}$ and $s_{\min}$ and $s_{\max}$ are the lower and upper bounds of the scale parameters, respectively. This term is used to smoothen $\tilde{g}_i(t)$ (see Note S3 for details). Here, $\frac{d\langle \boldsymbol{y}_i \rangle_+}{dt}$ denotes the nonnegative part of numerical derivative of $\boldsymbol{y}_i$ after applying moving average with the window size of $L = 7$. The second term $D_{\text{KL}}(q_{\theta_{\text{enc}}}(\boldsymbol{z}|\boldsymbol{y}) \| \text{N}(\boldsymbol{0}, \boldsymbol{I}))$ is a typical regulation loss of VAE used to ensure informative representation of data with latent variables (Figure S5).

Inference using Density-PINNs

After training Density-PINNs, we obtained the mean and standard deviation, $(\boldsymbol{\mu}, \boldsymbol{\sigma})$, for the latent variable $\boldsymbol{z}$ by passing the average of time traces $\overline{\boldsymbol{y}} = \frac{\sum_{i=1}^{\text{N}} \boldsymbol{y}_i}{N}$ through the encoder. Using $(\boldsymbol{\mu}, \boldsymbol{\sigma})$, we generated 1,000 latent samples $(\boldsymbol{z}^{(1)}, \boldsymbol{z}^{(2)}, \ldots, \boldsymbol{z}^{(1{,}000)})$ from a normal distribution $\text{N}(\boldsymbol{\mu}, \text{diag}(\boldsymbol{\sigma}^2))$. Each sample, $\boldsymbol{z}^{(l)}$ was then passed through the decoder, resulting in $(\boldsymbol{\omega}^{(l)}, \boldsymbol{s}^{(l)}, \lambda_d^{(l)}, \lambda_d^{(l)})$. With these parameters, we constructed an estimated transduction-time distribution $\tilde{g}^{(l)}(t)$ as follows:

$$\tilde{g}^{(l)}(t) = \sum_{j=1}^{M} \omega^{(l)}(c_j) K\left(t; c_j, s^{(l)}(c_j)\right).$$

We calculated the sample mean $m_{\tilde{g}}(t)$ and standard deviation $S_{\tilde{g}}(t)$ of $\{\tilde{g}^{(1)}(t), \tilde{g}^{(2)}(t), \ldots, \tilde{g}^{(1000)}(t)\}$. We then obtain the upper boundary $m_{\tilde{g}}(t) + 1.96 S_{\tilde{g}}(t)$ and the lower boundary $\max\{m_{\tilde{g}}(t) - 1.96 S_{\tilde{g}}(t), 0\}$, i.e., 95% prediction interval. The shaded regions in Figures 3B and 4B indicate the areas between the boundaries.

Interpretation of the response time, $\log(\lambda_b^{-1}) + \log(\mu_g)$

The inverse of activation rate λ_b (λ_b^{-1}) represents the signal initiation time per each molecule, as the unit of λ_b is the number of molecules per time. However, the unit of observed response molecules is often not the number of molecules but the unit of fluorescence. In this case, the estimated λ_b^{-1} has the following relationship with the true initiation time $\lambda_{b,\text{true}}^{-1}$: $\lambda_{b,\text{true}}^{-1} = \lambda_b^{-1} \times \gamma$ where the γ is a conversion rate from the number of molecules to the unit of fluorescence. Thus, to convert λ_b^{-1} to $\lambda_{b,\text{true}}^{-1}$, we need γ, which can be obtained by measuring the binomial error in partition of the total YFP signal during cell division.[66] However, γ is unknown in the experimental data we used in Figure 4. Therefore, we used $\log(\lambda_b^{-1}) + \log(\mu_g) = \log(\lambda_{b,\text{true}}^{-1}) + \log(\mu_g) - \log(\gamma)$ in Figure 4F. Since this is the sum of signal initiation time and transduction times in log scale, shifted by $\log(\gamma)$ (i.e., shifted response time in log scale), the observed positive associations in Figure 4F are preserved for the true response time without shift.

SUPPLEMENTAL INFORMATION

Supplemental information can be found online at https://doi.org/10.1016/j.patter.2023.100899.

ACKNOWLEDGMENTS

We thank Life Science Editors for editorial support and Sunghwan Bae (Bstar Artwork) for scientific illustration. H.H. is supported by the National Research Foundation of Korea (NRF) NRF-2019-Fostering Core Leaders of the Future Basic Science Program/Global PhD Fellowship Program 2019H1A2A1075303. H.J.H. is supported by the National Research Foundation of Korea (NRF) grant funded by the Korean government (MSIT) (no. RS-2022-00165268, RS-2023-00219980). W.C. is supported by the Charles Phelps Taft Research Center at the University of Cincinnati (no. M80941). J.K.K. is supported by the Institute for Basic Science IBS-R029-C3 and Samsung Science and Technology Foundation SSTF-BA1902-01.

AUTHOR CONTRIBUTIONS

H.J., H.H., H.J.H., and J.K.K. designed the study. H.J. developed an algorithm. H.J., H.H., W.C., and J.K.K. analyzed results. H.J., H.H., and J.K.K. wrote the manuscript. All authors revised the manuscript.

DECLARATION OF INTERESTS

The authors declare no competing interests.

Received: August 8, 2023
Revised: November 5, 2023
Accepted: November 24, 2023
Published: December 26, 2023

REFERENCES

1. Raj, A., and Van Oudenaarden, A. (2008). Nature, nurture, or chance: stochastic gene expression and its consequences. Cell *135*, 216–226.
2. Mitosch, K., Rieckh, G., and Bollenbach, T. (2019). Temporal order and precision of complex stress responses in individual bacteria. Mol. Syst. Biol. *15*, e8470.
3. Mitosch, K., Rieckh, G., and Bollenbach, T. (2017). Noisy response to antibiotic stress predicts subsequent single-cell survival in an acidic environment. Cell Syst. *4*, 393–403.e5.
4. Niepel, M., Spencer, S.L., and Sorger, P.K. (2009). Non-genetic cell-to-cell variability and the consequences for pharmacology. Curr. Opin. Chem. Biol. *13*, 556–561.
5. Kaern, M., Elston, T.C., Blake, W.J., and Collins, J.J. (2005). Stochasticity in gene expression: from theories to phenotypes. Nat. Rev. Genet. *6*, 451–464.
6. Lidstrom, M.E., and Konopka, M.C. (2010). The role of physiological heterogeneity in microbial population behavior. Nat. Chem. Biol. *6*, 705–712.
7. Tripathi, J., Zhu, L., Nayak, S., Stoklasa, M., and Bozdech, Z. (2022). Stochastic expression of invasion genes in Plasmodium falciparum schizonts. Nat. Commun. *13*, 3004.
8. Aymoz, D., Wosika, V., Durandau, E., and Pelet, S. (2016). Real-time quantification of protein expression at the single-cell level via dynamic protein synthesis translocation reporters. Nat. Commun. *7*, 11304–11312.
9. Phillips, N.E., Mandic, A., Omidi, S., Naef, F., and Suter, D.M. (2019). Memory and relatedness of transcriptional activity in mammalian cell lineages. Nat. Commun. *10*, 1208.
10. Roux, J., Hafner, M., Bandara, S., Sims, J.J., Hudson, H., Chai, D., and Sorger, P.K. (2015). Fractional killing arises from cell-to-cell variability in overcoming a caspase activity threshold. Mol. Syst. Biol. *11*, 803.
11. Wakamoto, Y., Dhar, N., Chait, R., Schneider, K., Signorino-Gelo, F., Leibler, S., and McKinney, J.D. (2013). Dynamic persistence of antibiotic-stressed mycobacteria. Science *339*, 91–95.
12. Wallden, M., Fange, D., Lundius, E.G., Baltekin, Ö., and Elf, J. (2016). The Synchronization of Replication and Division Cycles in Individual E. coli Cells. Cell *166*, 729–739.
13. Eling, N., Morgan, M.D., and Marioni, J.C. (2019). Challenges in measuring and understanding biological noise. Nat. Rev. Genet. *20*, 536–548.
14. Topolewski, P., Zakrzewska, K.E., Walczak, J., Nienałtowski, K., Müller-Newen, G., Singh, A., and Komorowski, M. (2022). Phenotypic variability, not noise, accounts for most of the cell-to-cell heterogeneity in IFN-gamma and oncostatin M signaling responses. Sci. Signal. *15*, eabd9303.
15. Zechner, C., Unger, M., Pelet, S., Peter, M., and Koeppl, H. (2014). Scalable inference of heterogeneous reaction kinetics from pooled single-cell recordings. Nat. Methods *11*, 197–202.
16. Di Talia, S., Skotheim, J.M., Bean, J.M., Siggia, E.D., and Cross, F.R. (2007). The effects of molecular noise and size control on variability in the budding yeast cell cycle. Nature *448*, 947–951.
17. Yang, S., Kim, S., Lim, Y.R., Kim, C., An, H.J., Kim, J.-H., Sung, J., and Lee, N.K. (2014). Contribution of RNA polymerase concentration variation to protein expression noise. Nat. Commun. *5*, 1–9.
18. Granados, A.A., Crane, M.M., Montano-Gutierrez, L.F., Tanaka, R.J., Voliotis, M., and Swain, P.S. (2017). Distributing tasks via multiple input pathways increases cellular survival in stress. Elife *6*, e21415.
19. Kalita, M.K., Sargsyan, K., Tian, B., Paulucci-Holthauzen, A., Najm, H.N., Debusschere, B.J., and Brasier, A.R. (2011). Sources of cell-to-cell variability in canonical nuclear factor-kappaB (NF-kappaB) signaling pathway inferred from single cell dynamic images. J. Biol. Chem. *286*, 37741–37757.
20. Chepyala, S.R., Chen, Y.C., Yan, C.C.S., Lu, C.Y.D., Wu, Y.C., and Hsu, C.P. (2016). Noise propagation with interlinked feed-forward pathways. Sci. Rep. *6*, 23607.
21. Kim, D.W., Hong, H., and Kim, J.K. (2022). Systematic inference identifies a major source of heterogeneity in cell signaling dynamics: The rate-limiting step number. Sci. Adv. *8*, eabl4598.
22. Choi, B., Cheng, Y.Y., Cinar, S., Ott, W., Bennett, M.R., Josić, K., and Kim, J.K. (2020). Bayesian inference of distributed time delay in transcriptional and translational regulation. Bioinformatics *36*, 586–593.
23. Cortez, M.J., Hong, H., Choi, B., Kim, J.K., and Josić, K. (2021). Hierarchical Bayesian models of transcriptional and translational regulation processes with delays. Bioinformatics *38*, 187–195.
24. Hong, H., Cortez, M.J., Cheng, Y.-Y., Kim, H.J., Choi, B., Josić, K., and Kim, J.K. (2022). Inferring delays in partially observed gene regulatory networks. Preprint at bioRxiv.
25. Thurley, K., Wu, L.F., and Altschuler, S.J. (2018). Modeling Cell-to-Cell Communication Networks Using Response-Time Distributions. Cell Syst. *6*, 355–367.e5.
26. Jiang, Q., Fu, X., Yan, S., Li, R., Du, W., Cao, Z., Qian, F., and Grima, R. (2021). Neural network aided approximation and parameter inference of non-Markovian models of gene expression. Nat. Commun. *12*, 1–12.
27. Calderazzo, S., Brancaccio, M., and Finkenstädt, B. (2019). Filtering and inference for stochastic oscillators with distributed delays. Bioinformatics *35*, 1380–1387.
28. Briat, C., and Khammash, M. (2020). Ergodicity Analysis and Antithetic Integral Control of a Class of Stochastic Reaction Networks with Delays. SIAM J. Appl. Dyn. Syst. *19*, 1575–1608.
29. Raissi, M., Perdikaris, P., and Karniadakis, G.E. (2019). Physics-informed neural networks: A deep learning framework for solving forward and inverse problems involving nonlinear partial differential equations. J. Comput. Phys. *378*, 686–707.
30. Gupta, C., López, J.M., Azencott, R., Bennett, M.R., Josić, K., and Ott, W. (2014). Modeling delay in genetic networks: from delay birth-death processes to delay stochastic differential equations. J. Chem. Phys. *140*, 204108.
31. Josić, K., López, J.M., Ott, W., Shiau, L., and Bennett, M.R. (2011). Stochastic delay accelerates signaling in gene networks. PLoS Comput. Biol. *7*, e1002264.
32. Barrio, M., Leier, A., and Marquez-Lago, T.T. (2013). Reduction of chemical reaction networks through delay distributions. J. Chem. Phys. *138*, 104114.
33. Leier, A., Barrio, M., and Marquez-Lago, T.T. (2014). Exact model reduction with delays: closed-form distributions and extensions to fully bi-directional monomolecular reactions. J. R. Soc. Interface *11*, 20140108.
34. Hwang, H.J., Jang, J.W., Jo, H., and Lee, J.Y. (2020). Trend to equilibrium for the kinetic Fokker-Planck equation via the neural network approach. J. Comput. Phys. *419*, 109665.
35. Cavanagh, H., Mosbach, A., Scalliet, G., Lind, R., and Endres, R.G. (2021). Physics-informed deep learning characterizes morphodynamics of Asian soybean rust disease. Nat. Commun. *12*, 6424.
36. Gayoso, A., Steier, Z., Lopez, R., Regier, J., Nazor, K.L., Streets, A., and Yosef, N. (2021). Joint probabilistic modeling of single-cell multi-omic data with totalVI. Nat. Methods *18*, 272–282.
37. Chen, M., and Ludtke, S.J. (2021). Deep learning-based mixed-dimensional Gaussian mixture model for characterizing variability in cryo-EM. Nat. Methods *18*, 930–936.
38. Cai, X. (2007). Exact stochastic simulation of coupled chemical reactions with delays. J. Chem. Phys. *126*, 124108.
39. Andersen, J.B., Sternberg, C., Poulsen, L.K., Bjørn, S.P., Givskov, M., and Molin, S. (1998). New unstable variants of green fluorescent protein for studies of transient gene expression in bacteria. Appl. Environ. Microbiol. *64*, 2240–2246.

CellPress
OPEN ACCESS

40. Rosenfeld, N., Elowitz, M.B., and Alon, U. (2002). Negative autoregulation speeds the response times of transcription networks. J. Mol. Biol. *323*, 785–793.
41. Becskei, A., and Serrano, L. (2000). Engineering stability in gene networks by autoregulation. Nature *405*, 590–593.
42. Alon, U. (2007). Network motifs: theory and experimental approaches. Nat. Rev. Genet. *8*, 450–461.
43. Gewandter, J.S., McDermott, M.P., He, H., Gao, S., Cai, X., Farrar, J.T., Katz, N.P., Markman, J.D., Senn, S., Turk, D.C., and Dworkin, R.H. (2019). Demonstrating heterogeneity of treatment effects among patients: an overlooked but important step toward precision medicine. Clin. Pharmacol. Ther. *106*, 204–210.
44. Schnoerr, D., Sanguinetti, G., and Grima, R. (2015). Comparison of different moment-closure approximations for stochastic chemical kinetics. J. Chem. Phys. *143*, 185101.
45. Zhang, Q., Bhattacharya, S., and Andersen, M.E. (2013). Ultrasensitive response motifs: basic amplifiers in molecular signalling networks. Open Biol. *3*, 130031.
46. Karniadakis, G.E., Kevrekidis, I.G., Lu, L., Perdikaris, P., Wang, S., and Yang, L. (2021). Physics-informed machine learning. Nat. Rev. Phys. *3*, 422–440.
47. Okazaki, T., Ito, T., Hirahara, K., and Ueda, N. (2022). Physics-informed deep learning approach for modeling crustal deformation. Nat. Commun. *13*, 7092.
48. Sukys, A., Öcal, K., and Grima, R. (2022). Approximating solutions of the Chemical Master equation using neural networks. iScience *25*, 105010.
49. Beentjes, C.H.L., Perez-Carrasco, R., and Grima, R. (2020). Exact solution of stochastic gene expression models with bursting, cell cycle and replication dynamics. Phys. Rev. E *101*, 032403.
50. Krishnapriyan, A., Gholami, A., Zhe, S., Kirby, R., and Mahoney, M.W. (2021). Characterizing possible failure modes in physics-informed neural networks. Preprint at arXiv. https://doi.org/10.48550/arXiv.2109.01050s.
51. Wang, S., Yu, X., and Perdikaris, P. (2022). When and why PINNs fail to train: A neural tangent kernel perspective. J. Comput. Phys. *449*, 110768.
52. Goswami, S., Kontolati, K., Shields, M.D., and Karniadakis, G.E. (2022). Deep transfer operator learning for partial differential equations under conditional shift. Nat. Mach. Intell. *4*, 1155–1164.
53. Li, R., Wang, J.X., Lee, E., and Luo, T. (2022). Physics-informed deep learning for solving phonon Boltzmann transport equation with large temperature non-equilibrium. npj Comput. Mater. *8*, 29.
54. Kharazmi, E., Cai, M., Zheng, X., Zhang, Z., Lin, G., and Karniadakis, G.E. (2021). Identifiability and predictability of integer-and fractional-order epidemiological models using physics-informed neural networks. Nat. Comput. Sci. *1*, 744–753.
55. Jung, S.Y., Jo, H., Son, H., and Hwang, H.J. (2020). Real-world implications of a rapidly responsive COVID-19 spread model with time-dependent parameters via deep learning: Model development and validation. J. Med. Internet Res. *22*, e19907.
56. Zhang, D., Lu, L., Guo, L., and Karniadakis, G.E. (2019). Quantifying total uncertainty in physics-informed neural networks for solving forward and inverse stochastic problems. J. Comput. Phys. *397*, 108850.
57. Zhang, D., Guo, L., and Karniadakis, G.E. (2020). Learning in modal space: Solving time-dependent stochastic PDEs using physics-informed neural networks. SIAM J. Sci. Comput. *42*, A639–A665.
58. Lu, L., Jin, P., Pang, G., Zhang, Z., and Karniadakis, G.E. (2021). Learning nonlinear operators via DeepONet based on the universal approximation theorem of operators. Nat. Mach. Intell. *3*, 218–229.
59. Mbuvha, R., and Marwala, T. (2020). Bayesian inference of COVID-19 spreading rates in South Africa. PLoS One *15*, e0237126.
60. Skinner, D.J., and Dunkel, J. (2021). Estimating entropy production from waiting time distributions. Phys. Rev. Lett. *127*, 198101.
61. Skinner, D.J., and Dunkel, J. (2021). Improved bounds on entropy production in living systems. Proc. Nat. Acad. Sci. USA *118*, e2024300118.
62. Jo, H., Hong, H., Hwang, H.J., Chang, W., and Kim, J.K. (2023). DensityPINNs (Zenodo). https://doi.org/10.5281/zenodo.10108680.
63. Kingma, D.P., and Ba, J. (2014). Adam: A method for stochastic optimization. Preprint at arXiv. https://doi.org/10.48550/arXiv.1412.6980.
64. Kingma, D.P., and Welling, M. (2013). Auto-encoding variational bayes. Preprint at arXiv. https://doi.org/10.48550/arXiv.1312.6114.
65. Baydin, A.G., Pearlmutter, B.A., Radul, A.A., and Siskind, J.M. (2018). Automatic Differentiation in Machine Learning: a Survey. Preprint at arXiv.
66. Rosenfeld, N., Young, J.W., Alon, U., Swain, P.S., and Elowitz, M.B. (2005). Gene regulation at the single-cell level. Science *307*, 1962–1965.

Patterns, Volume *5*

Supplemental information

Density physics-informed neural networks reveal sources of cell heterogeneity in signal transduction

Hyeontae Jo, Hyukpyo Hong, Hyung Ju Hwang, Won Chang, and Jae Kyoung Kim

SUPPLEMENTAL INFORMATION

Supplemental Notes

Note S1. Differential equation describing the mean of a stochastic delayed birth-death process

A stochastic delayed birth-death process can be used to describe a cell signaling pathway that consists of signal activation, transduction, and final response (Figure 1A). Specifically, signal activation is represented by a birth reaction with a rate of λ_b, the activated signal undergoes a delay with density $g(t)$, and the decay of the final response molecules is represented by a death reaction with a rate of λ_d. According to the Transient Little's law,[1] the mean time trace of the stochastic process $y(t)$, is given by

$$y(t) = \int_0^t \lambda_b G(t-\tau)\exp(-\lambda_d \tau)\, d\tau = \lambda_b (G * h)(t),$$

where $G(t) = \int_0^t g(s)ds$ and $h(t) = \exp(-\lambda_d t)$ for $t \geq 0$ and $h(t) = 0$ for $t < 0$, which represents a survival probability of the response molecules after time t (see Kim et al.[2] for details). To derive the differential equation for $y(t)$, we took a derivative and got the following:

$$y'(t) = \lambda_b (G * h')(t) = \lambda_b (G * [\delta_0(\tau) + (-\lambda_d) h])(t) = \lambda_b G(t) - \lambda_d \lambda_b (G * h)(t)$$

where $\delta_0(\tau)$ is the Dirac delta measure at time 0. Finally, we obtained the differential equation that the mean time trace $y(t)$ satisfies as follows:

$$y'(t) = \lambda_b \int_0^t g(s)ds - \lambda_d y(t).$$

This differential equation is used to form a physics loss for our inference model (Figure 2B).

Note S2. Minimizing the data and physics losses ensures the convergence of an approximated transduction-time distribution, $\tilde{g}(t)$, to the true transduction-time distribution, $g(t)$.

We use $\tilde{y}(t) = \sum_{j=1}^{M} \omega_j' \int_0^t K(u; c_j, s_j) du$ instead of the exact solution of Equation 1, $\tilde{y}_{ex}(t) = \lambda_b \sum_{j=1}^{M} \omega_j e^{-\lambda_d t} * \int_0^t K(u; c_j, s_j) du$ for computational efficiency (Figure 2). Here, we justify this by showing that even when $\tilde{y}(t)$ is used, $\tilde{g}$ converges to g when $\tilde{y}(t)$ minimizes both data and physics losses simultaneously. To do this, we prove the following two steps.

Step 1: $\tilde{y}_{ex}(t)$ converges to y when $\tilde{y}(t)$ minimizes both data and physics losses.
Step 2: $\tilde{g}$ converges to g when $\tilde{y}_{ex}$ converges to y.

Proof of Step 1: We indirectly quantify the difference between $\tilde{y}_{ex}$ and $\tilde{y}$ using the physics loss. First, let $p(t)$ be the function given by substituting $\tilde{y}$ into Equation 1.

$$p(t) := \frac{d\tilde{y}}{dt} + \lambda_d \tilde{y} - \lambda_b \int_0^t \tilde{g}(u)du.$$

Since $\tilde{y}_{ex}$ exactly satisfies the equation $\frac{d\tilde{y}_{ex}}{dt} + \lambda_d \tilde{y}_{ex} = \lambda_b \int_0^t \tilde{g}(u)du$, we can replace $\int_0^t \tilde{g}(u)du$ with $\frac{d\tilde{y}_{ex}}{dt} + \lambda_d \tilde{y}_{ex}$ as follows:

$$p(t) = \frac{d}{dt}(\tilde{y} - \tilde{y}_{ex}) + \lambda_d (\tilde{y} - \tilde{y}_{ex}).$$

By solving the differential equation with respect to $\tilde{y} - \tilde{y}_{ex}$, we obtain the following formula:

$$\tilde{y} - \tilde{y}_{ex} = e^{-\lambda_d t} * p(t).$$

By taking $L^1([0,T])$-norm on both sides and using the Young's convolution inequality, we obtain

$$\|\tilde{y} - \tilde{y}_{ex}\|_{L^1([0,T])} \leq \left\|e^{-\lambda_d t}\right\|_{L^1([0,T])} \|p(t)\|_{L^1([0,T])}.$$

Thus, $\tilde{y}$ converges to $\tilde{y}_{ex}$ by minimizing the physic loss, $\|p(t)\|_{L^1([0,T])}$. Finally, using the triangle inequality,

$$\|\tilde{y}_{ex} - y\|_{L^1([0,T])} \leq \|\tilde{y}_{ex} - \tilde{y}\|_{L^1([0,T])} + \|\tilde{y} - y\|_{L^1([0,T])}$$

$$\leq \left\| e^{-\lambda_d t} \right\|_{L^1([0,T])} \text{physics loss} + \text{data loss},$$

we can conclude that $\tilde{y}$ can closely approximate $\tilde{y}_{ex}$ by simultaneously minimizing both data and physics losses. We also showed this using numerical optimization (Figure S1).

Proof of Step2: y and $\tilde{y}_{ex}$ satisfy Equation 1 corresponding to g and $\tilde{g}$, respectively. That is,

$$\frac{d\tilde{y}_{ex}}{dt} + \lambda_d \tilde{y}_{ex} = \lambda_b \int_0^t \tilde{g}(u) du,$$

$$\frac{dy}{dt} + \lambda_d y = \lambda_b \int_0^t g(u) du.$$

By substituting both sides and taking $L^1([0,T])$-norm, we can derive the following inequality:

$$\lambda_b \left\| \int_0^t \left(\tilde{g}(u) - g(u)\right) du \right\|_{L^1([0,T])} = \|(\tilde{y}_{ex} - y)' + \lambda_d (\tilde{y}_{ex} - y)\|_{L^1([0,T])}$$

$$\leq \|(\tilde{y}_{ex} - y)'\|_{L^1([0,T])} + \lambda_d \|\tilde{y}_{ex} - y\|_{L^1([0,T])}.$$

We then aim to show $\|(\tilde{y}_{ex} - y)'\|_{L^1([0,T])}$ is close to 0 when $\|\tilde{y}_{ex} - y\|_{L^1([0,T])}$ is close to 0. Let $\tilde{y}_{ex,n}$ and $\tilde{g}_n$ denote the trained functions at n-th iteration when we minimize $\|\tilde{y}_{ex} - y\|_{L^1([0,T])}$, i.e., $\tilde{g}_n = \sum_{j=1}^{M} \omega_{j,n} K\left(t; c_{j,n}, s_{j,n}\right)$, for $n = 1,2, \ldots$. It is clear that $\tilde{y}_{ex,n}$ is bounded by the steady state solution $\frac{\lambda_b}{\lambda_d}$, i.e., $\tilde{y}_{ex,n}(t) \leq \frac{\lambda_b}{\lambda_d}$, for any $\tilde{g}_n$ and time $t \in [0,T]$. Then, we obtain the following upper bound for $\tilde{y}'_{ex,n}(t)$:

$$\left|\tilde{y}'_{ex,n}(t)\right| \leq \lambda_d \left|\tilde{y}_{ex,n}(t)\right| + \lambda_b \left| \int_0^t \tilde{g}_n(u) du \right| \leq \lambda_d \frac{\lambda_b}{\lambda_d} + \lambda_b = 2\lambda_b.$$

By substituting the differential equations for $\tilde{y}_{ex,n}(t)$ and $\tilde{y}_{ex,n}(v)$, for $t, v \in [0,T]$, we can derive the following inequality:

$$\left|\tilde{y}'_{ex,n}(t) - \tilde{y}'_{ex,n}(v)\right| \leq \lambda_d \left|\tilde{y}_{ex,n}(t) - \tilde{y}_{ex,n}(v)\right| + \lambda_b \left| \int_v^t \tilde{g}_n(u) du \right|$$

$$\leq 2\lambda_b \lambda_d |t - v| + \lambda_b C(s_1, \ldots, s_M)|t - v|,$$

where the first term in the last inequality is obtained by the mean-value theorem, and the $C(s_1, \ldots, s_M)$ is the Lipschitz constant for $\int_v^t \tilde{g}_n(u) du$. Since $C(s_1, \ldots, s_M)$ is independent of (n, t, v) and s_j in $C(s_1, \ldots, s_M)$ is contained in $[s_{min}, s_{max}]$, $\left|\tilde{y}'_{ex,n}(t)\right|$ is Lipschitz continuous with a constant $C = 2\lambda_b \lambda_d + \lambda_b C(s_1, \ldots, s_M)$. That is,

$$\left|\tilde{y}'_{ex,n}(t) - \tilde{y}'_{ex,n}(v)\right| \leq C|t - v|,$$

which implies that $\left\{\tilde{y}_{ex,n}\right\}_{n=1}^{\infty}$ is equicontinuous. Since $\left\{\tilde{y}'_{ex,n}\right\}_{n=1}^{\infty}$ is uniformly bounded and equicontinuous, we can extract a uniformly convergent subsequence by the Arzela-Ascoli theorem, and its limit converges to y'. In summary, if $\tilde{y}_{ex}$ converges to y during the training step, $\int_0^t \tilde{g}(u) du$ converges to $\int_0^t g(u) du$. Consequently, $\tilde{g}(t)$ converges to $g(t)$ by differentiating Equation 1 and by repeating same procedure.

Note S3. $\|\boldsymbol{s}(\boldsymbol{\theta}_{\text{enc}}, \boldsymbol{\theta}_{\text{dec}})\|$ in the regularization loss ensures the smoothness of an estimated transduction-time distribution $\tilde{\boldsymbol{g}}(\boldsymbol{t})$.

In the regularization loss $\mathcal{L}_r(\theta) = \|s(\theta_{\text{enc}}, \theta_{\text{dec}})\| + D_{\text{KL}}\left(q_{\theta_{\text{enc}}}(\boldsymbol{z}|\boldsymbol{y}) || N(\boldsymbol{0}, \boldsymbol{I})\right)$ (Figure 2B), the first term is given by

$$\|s(\theta_{\text{enc}}, \theta_{\text{dec}})\| = \frac{1}{Nd} \sum_{i=1}^{N} \sum_{j=1}^{d} \left| s_i(t_j) - \left(\beta_i(t_j) s_{\min} + \left(1 - \beta_i(t_j)\right) s_{\max}\right) \right|,$$

where $\beta_i(t_j) = \frac{\frac{d\langle y_i \rangle_+}{dt}(t_j) - \min \frac{d\langle y_i \rangle_+}{dt}(t)}{\max \frac{d\langle y_i \rangle_+}{dt}(t) - \min \frac{d\langle y_i \rangle_+}{dt}(t)}$ and $s_{\min}$ and $s_{\max}$ are the lower and upper bounds of the scale parameters, respectively. This term ensures the smoothness of an estimated transduction-time distribution $\tilde{g}(t)$ by preventing too small or large values of the scale parameters. Specifically, when

a time trace $\boldsymbol{y}_i$ dramatically changes, i.e., $\frac{d\langle y_i\rangle_+}{dt}(t_j) \approx \max\frac{d\langle y_i\rangle_+}{dt}(t)$, we get $\beta_i(t_j) \approx 1$ and thus $s_i(t_j) \approx s_{\min}$. That is, when there is a dramatic change in $\boldsymbol{y}_i$, the scale parameters are set to be small and thus the width of the kernel at t_j becomes narrow. On the other hand, when the time trace $\boldsymbol{y}_i$ is nearly flat, i.e., $\frac{d\langle y_i\rangle_+}{dt}(t_j) \approx \min\frac{d\langle y_i\rangle_+}{dt}(t)$, we get $\beta_i(t_j) \approx 0$, and hence $s_i(t_j)$ is penalized if it is far from $s_{\max}$. That is, when there is no dramatic change in $\boldsymbol{y}_i$, we encourage the scale parameters to be large so that the kernel with a large width is used. In this way, we can avoid the use of sharp kernels, which prevents redundant fluctuation in the $\tilde{g}(t)$. That is, with the regularization term $\|s(\theta_{\text{enc}}, \theta_{\text{dec}})\|$, we can get smooth $\tilde{g}(t)$.

Note S4. Stopping criterion for training Density-PINN.

We trained the model using the Adam optimizer[3] with learning rate = 10^{-4}. In order to prevent overfitting issues, we applied an early stopping criterion[4]. During the training process, we generated 1000 samples of jth weight $\omega(c_j)$ from $p_{\theta_{\text{dec}}}\left(\boldsymbol{\omega}|q_{\theta_{\text{enc}}}(\boldsymbol{z}|\bar{\boldsymbol{y}})\right)$ at l^{th} epoch where $\bar{\boldsymbol{y}}$ is the average of time traces. We measured the average value for $\omega(c_j)$ at the lth epoch, $\bar{\omega}(c_j)[l]$. Subsequently, we computed the coefficient of variation for $\bar{\omega}(c_j)[l]$ among the last h epochs, i.e., from $(n-h+1)^{th}$ to n^{th} epoch. That is,

$$cv\left(\bar{\omega}(c_j)\right) \coloneqq cv\left(\bar{\omega}(c_j)\right)[n-h+1{:}n] = \frac{std\left(\bar{\omega}(c_j)\right)[n-h+1{:}n]}{mean\left(\bar{\omega}(c_j)\right)[n-h+1{:}n]+\varepsilon}.$$

Among $cv(\bar{\omega}(c_1)), \ldots, cv(\bar{\omega}(c_M))$, if the number of $cv\left(\bar{\omega}(c_j)\right)$'s less than the threshold=0.01 reaches 99%, we stopped training the model. Note that h was set to be 30, and $\varepsilon = 10^{-5}$ was added in the denominator since some weights can be close to zero during training.

Note S5. Stochastic simulation

We used a delayed stochastic simulation algorithm[5] to generate the simulation data used to validate our method (Figure 3). Specifically, we performed simulation for the birth-death process with delays on the birth reaction completion[6]. The birth rate $\lambda_b = 200$, and the death rate $\lambda_d = 1$. For unimodal, weakly bimodal, strongly bimodal distributions, the PDF of the delay distributions are given by $f_\Gamma(t; 3{,}0.6)$, $0.7f_\Gamma(t; 4.3{,}0.4) + 0.3f_\Gamma(t-1.6; 15{,}0.15)$, $0.6f_\Gamma(t; 4.3{,}0.4) + 0.4f_\Gamma(t-1.6; 15{,}0.15)$ where $f_\Gamma(t; a, b)$ is the PDF of Gamma distribution with the shape parameter a and the scale parameter b.

Note S6. Dip statistic for multimodality

A multimodal distribution is a statistical distribution with more than one peak in its probability density function (PDF). The PDF of a multimodal distribution has multiple points where it transitions from increasing to decreasing or vice versa. Thus, the corresponding cumulative distribution function (CDF) of a multimodal distribution has multiple inflection points, where it changes from being convex to concave or vice versa. The magnitude of the multimodality can be quantified with the maximum distance between the given multimodal CDF F and its closest unimodal CDF, known as the dip statistic[7]:

$$Dip(F) = \inf_{G\in\mathcal{A}} \sup_x |F(x) - G(x)|,$$

where $\mathcal{A}$ is the class of all unimodal distribution functions.

Note S7. Step-by-step manual for Density-PINN.

We have developed a computational package implementing Density-PINN that infers delay distributions in stochastic processes. The Python code, written in Jupyter, is available at https://Github.com/mathbiomed/Density-PINN (This link will be public upon acceptance, please see the submitted code files.)

Step 1. Upload time trace data CSV file to the 'data' folder. The first column of the CSV file should contain observation time points, and the column name should be entered in the first row. For the nth row ($n \geq 2$), fill in the time series data with the name of each column. An example input file named 'sample_data.csv' is included in the 'data' folder for testing our model in Figure 3B. Ensure that the name of each column is unique.

Step 2. Open 'Density-PINN.ipynb' using Jupyter notebook. In the second cell, specify all the hyperparameters listed in Table S2 and set the 'data_name' variable to the name of your input CSV file. After making these changes, run the code by clicking 'Restart and Run All'. The trained model will be saved in the 'model' folder with the same name as the CSV file, and the program will automatically close upon completion of training.

(optional). Customize your own governing equation or target variable by editing 'utils.py' file Line 66. This file contains the model architecture and optimization code implemented using *PyTorch*.

Step 3. Run 'Visualization.ipynb'. The below output file in 'result' folder will be automatically created.

'estimation.png' visualizes an estimated transduction-time distribution and parameters, λ_b and λ_d. It also provides a comparison between the mean of time traces and the estimated $\tilde{y}(t)$ from the trained Density-PINN.

Supplemental Figures

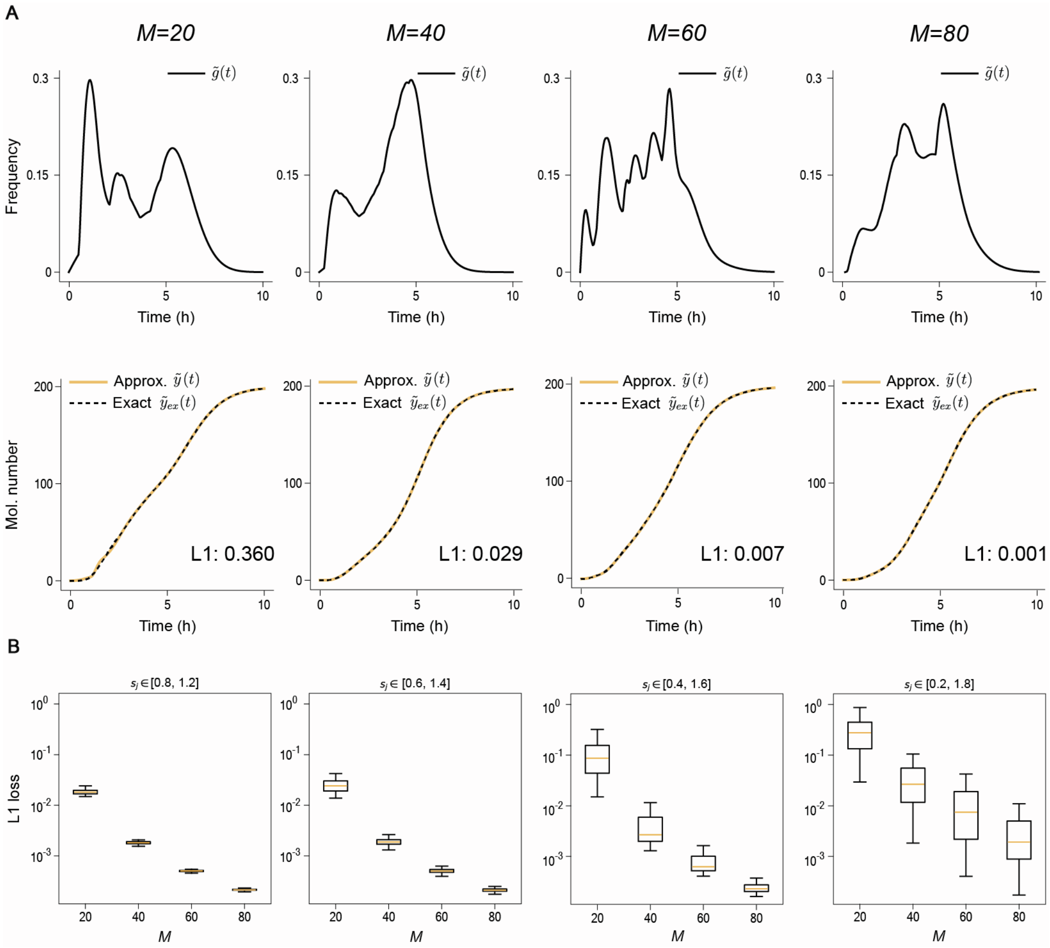

Figure S1. An approximate solution $\tilde{y}(t)$ can accurately estimate an exact solution $\tilde{y}_{ex}(t)$ of Equation 1 with $\tilde{g}(t)$. (A) We generated $\tilde{g}(t) := \sum_{j=1}^{M} \omega_j K(t; c_j, s_j)$ using M shifted Rayleigh kernels with the shift and scale parameters c_j and s_j, respectively, and the weights ω_j. Here, $c_j = \frac{10}{2(M-1)}(j-1)$, s_j and ω_j were uniformly sampled from [0.2, 1.8] and [0,1], respectively, and ω_j were normalized so that their sum is one (top). For a given $\tilde{g}(t)$, we constructed the corresponding exact solution $\tilde{y}_{ex}(t) = \lambda_b \sum_{j=1}^{M} \omega_j e^{-\lambda_d t} * \int_0^t K(u; c_j, s_j)du$ of Equation 1 and calculated ω_j' for $\tilde{y}(t) = \sum_{j=1}^{M} \omega_j' \int_0^t K(u; c_j, s_j)du$ using the Levenberg-Marquardt method so that $\tilde{y}(t)$ approximates $\tilde{y}_{ex}(t)$ (bottom). The accuracy of $\tilde{y}(t)$ in approximating $\tilde{y}_{ex}(t)$ is quantified with the L1 loss using 100 time points evenly space in the time domain $[0, 10]$. As M increases, $\tilde{y}(t)$ more accurately approximates $\tilde{y}(t)$. (B) We repeated this process 100 times for various range of the scale parameters: $s_j \in$ [0.8, 1.2], [0.6, 1.4], [0.4, 1.6], or [0.2, 1.8]. The L1 loss between $\tilde{y}_{ex}(t)$ and $\tilde{y}(t)$ decreased as M increased in all cases.

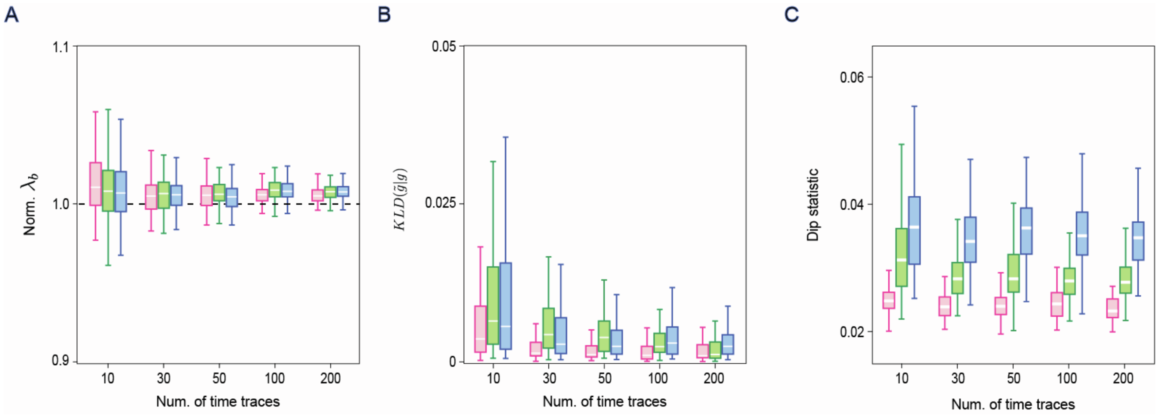

Figure S2. Density-PINN provides more accurate estimates of the activation rates and transduction-time distributions when the decay rate is fixed to its true value compared with when the decay rate is also estimated (Figure 3). (A-C) As more time traces were used for the inference, the estimation becomes more accurate: estimations of the λ_b become more accurate and precise (A) the KL-divergence between the underlying and reconstructed transduction-time distributions decreases (B), and the dip statistic, which increases as the bimodality increases, becomes more clearly distinguished among the unimodal (red), weakly bimodal (green), and strongly bimodal (blue) distributions (C).

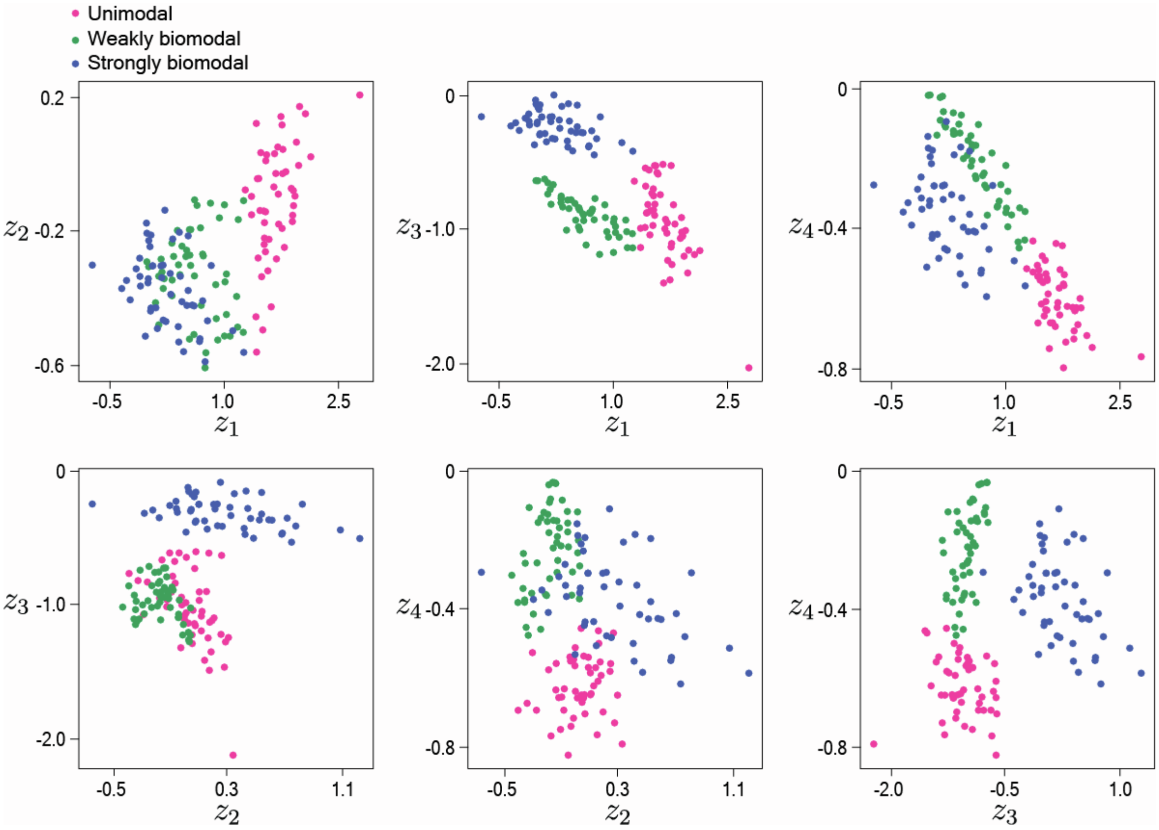

Figure S3. Visualization of latent variables corresponding to the time traces in Figure 3B. Density-PINN was trained using all time traces in Figure 3B. The resulting 4D latent variable, $\boldsymbol{z} = (z_1, z_2, z_3, z_4)$, was visualized by projecting into two dimensional spaces. The latent variables corresponding to time traces from three different types of transduction-time distributions were depicted as different colored dots: unimodal (red), weakly bimodal (green), and strongly bimodal (blue). The clear separation of the dots with different colors indicates that the Density-PINN can distinguish time traces from different transduction-time distributions.

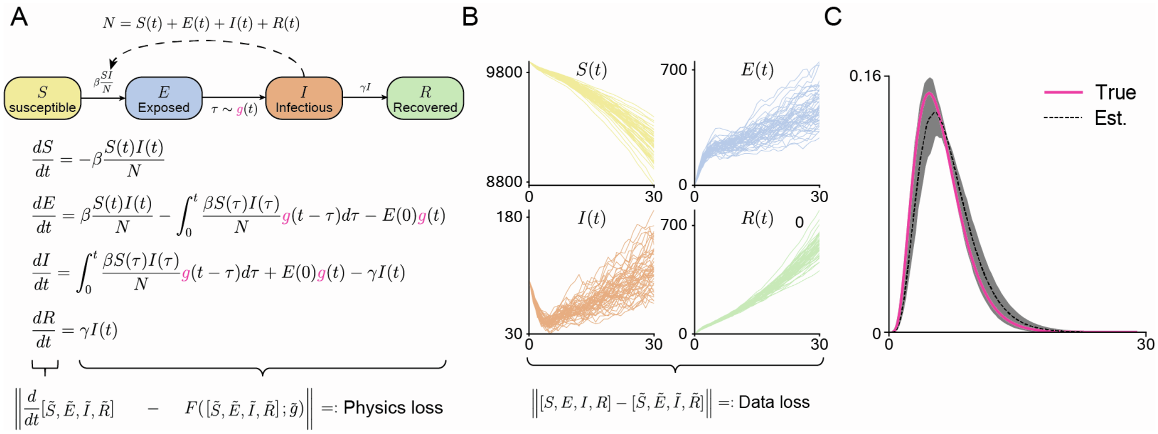

Figure S4. Density-PINN accurately estimates the latent period distribution of the SEIR Model. (A) The Susceptible-Exposed-Infectious-Recovered (SEIR) compartmental model with the latent period distribution $g(t)$ (i.e., the sojourn time in the compartment E). The dynamics of the model can be described by the system of differential equations. These equations were exploited to define a physics loss function for training of Density-PINN, where the original S, E, I, R, and g were substituted with corresponding neural network approximations: $\tilde{S}$, $\tilde{E}$, $\tilde{I}$, $\tilde{R}$, and $\tilde{g}$, respectively. (B) Through a delayed stochastic simulation algorithm[5], we produced 50 time traces of the compartments S, E, I, and R with the transmission rate $\beta = 0.35$, recovery rate $\gamma = 0.25$, and the latent period distribution $g(t) \sim \Gamma(5, 1)$. These traces were then employed to define a data loss by quantifying the distance between the given traces and neural networks. (C) We trained Density-PINN by minimizing both physics and data losses. After training, Density-PINN yields accurate estimates of $g(t)$, including prediction intervals (black). As this result was presented for illustration of scalability of Density-PINN to an example other than cell-signaling pathway, we have made several assumptions helping the estimation of $g(t)$. Specifically, we assumed that both β and γ are known and time traces of all compartments S, E , I , and R are observable. While these assumptions resolve identifiability issues when we estimate $g(t)$, it may not practical in real-world applications. Therefore, relaxing these assumptions would be an important future avenue of research to explore.

Supplemental Tables

Table S1. Abbreviations for the promoters' name

Promoter	Abbrebation
wrba	wa
dps	ds
gadw	gw
iscR	iR
gadb	gb
cspA	cA
rpmE	rE
purM	pM
purT	pT
nrdH	nH
ldha	la
ahpC	aC
purT	pT
gada	ga
osmc	oc
dnaK	dK
ydiU	yU

Table S2. Selection of hyperparameters.

Part	Name	Numb. of Nodes	Activation functions[1]	Input
Data	Input	$Input\_dim\ (d)$	-	-
Encoder	Hidden_ 1	16	$ELU(\,)$	Input
	Hidden_ 2	16	$ELU(\,)$	Hidden_1
Latent	mean	$Latent\_dim\ (k = 4)$	-	Hidden _2
	logvar	$k = 4$	-	Hidden _2
Decoder $\boldsymbol{\omega}$	Hidden_3	16	$ELU(\,)$	[mean, logvar]
	Hidden_4	16	$ELU(\,)$	Hidden_3
	$\boldsymbol{\omega}$	$N\_comp\ (M = 80)$	$Softmax(\,)$	Hidden_4
Decoder $\boldsymbol{s}$	Hidden_5	16	$ELU(\,)$	[mean, logvar]
	Hidden_6	16	$ELU(\,)$	Hidden_5
	$\boldsymbol{s}$	$M = 80$	$(s_{max} - s_{min}) \times Sigmoid(\,) + s_{min}$	Hidden_6
Decoder λ_b, λ_d	Hidden_7	16	$ELU(\,)$	[mean, logvar]
	λ_b, λ_d	2	$ELU(\,) + 1$	Hidden_7
NN	Hidden_8	$M = 80$	$ELU(\,)$	$\boldsymbol{\omega}$
	$\boldsymbol{\omega}'$	$M = 80$	$ELU(\,) + 1$	Hidden_8

[1]The following activation functions $ELU(x)$, $Sigmoid(x)$ and $Softmax(x)$ were used:

$$ELU(x_j) = x_j \text{ if } x_j > 0, \qquad \exp(x_j) - 1 \text{ otherwise,}$$

$$Sigmoid(x_j) = \frac{1}{1 + \exp(-x_j)},$$

$$Softmax(x_j) = \frac{e^{x_j}}{\sum_{k=1}^{J} e^{x_k}}.$$

Supplemental References

1. Bertsimas, D., and Mourtzinou, G. (1997). Transient laws of non-stationary queueing systems and their applications. Queueing Systems *25*, 115-155.
2. Kim, D.W., Hong, H., and Kim, J.K. (2022). Systematic inference identifies a major source of heterogeneity in cell signaling dynamics: The rate-limiting step number. Sci. Adv. *8*, eabl4598. 10.1126/sciadv.abl4598.
3. Kingma, D.P., and Ba, J. (2014). Adam: A method for stochastic optimization. arXiv preprint arXiv:1412.6980.
4. Kingma, D.P., and Welling, M. (2013). Auto-encoding variational bayes. arXiv preprint arXiv:1312.6114.
5. Cai, X. (2007). Exact stochastic simulation of coupled chemical reactions with delays. J Chem. Phys. *126*, 124108. 10.1063/1.2710253.
6. Gupta, C., Lopez, J.M., Azencott, R., Bennett, M.R., Josic, K., and Ott, W. (2014). Modeling delay in genetic networks: from delay birth-death processes to delay stochastic differential equations. J Chem. Phys. *140*, 204108. 10.1063/1.4878662.
7. Hartigan, J.A., and Hartigan, P.M. (1985). The dip test of unimodality. Ann.Stat., 70--84.
